# Supplementary material for: Ion-pair interactions between voltage-sensing domain IV and pore domain I regulate CaV1.1 gating
Source: Biophys J. 2021 Sep 8;120(20):4429–41. doi: 10.1016/j.bpj.2021.09.004 (PMC8553663; doi:10.1016/j.bpj.2021.09.004)
Supplement: Document S1. Figs. S1–S6 and Table S1 [file mmc1.pdf]

Biophysical Journal, Volume *120*

Supplemental information

Ion-pair interactions between voltage-sensing domain IV and pore domain I regulate $Ca_V1.1$ gating

Yousra El Ghaleb, Monica L. Fernández-Quintero, Stefania Monteleone, Petronel Tuluc, Marta Campiglio, Klaus R. Liedl, and Bernhard E. Flucher

A

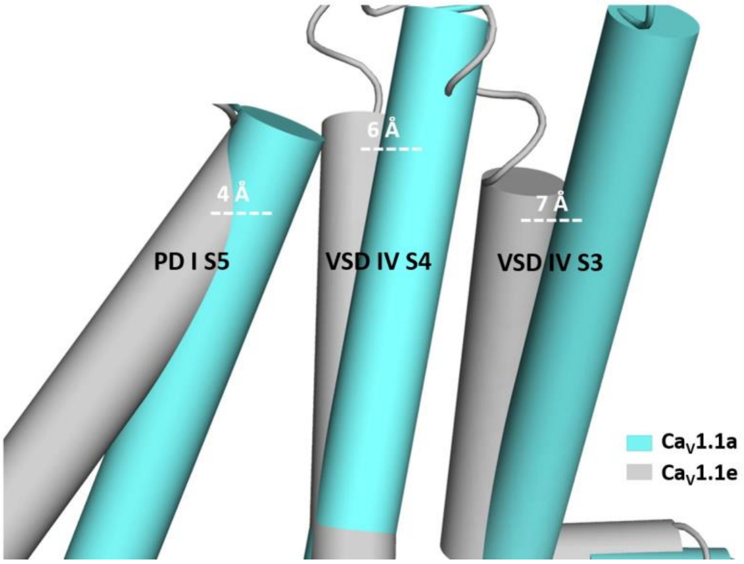

B

Cα-distance (Å)	Ca_V1.1a	Ca_V1.1e	Δ
**E216 - R1**	6.6	7.8	1.2
**D4 - R1**	6.4	5.4	1.0

Figure S1. Insertion of exon 29 in the S3-S4 linker of VSD IV causes a displacement of the α-helices and changes in the relative position of the Cα-atoms of the ion pair partners. (**A**) Cylinder-model showing IS5, IVS3 and IVS4 of Ca_V1.1e (lacking exon 29, gray) and Ca_V1.1a (including exon 29, turquoise) derived from overlays of the structure models. Displacement distances of the Cα-atoms of E216, R1, and D1196 are shown for the two splice variants. (**B**) Table showing the displacement and the resulting changes in the distance between the ion pair partners.

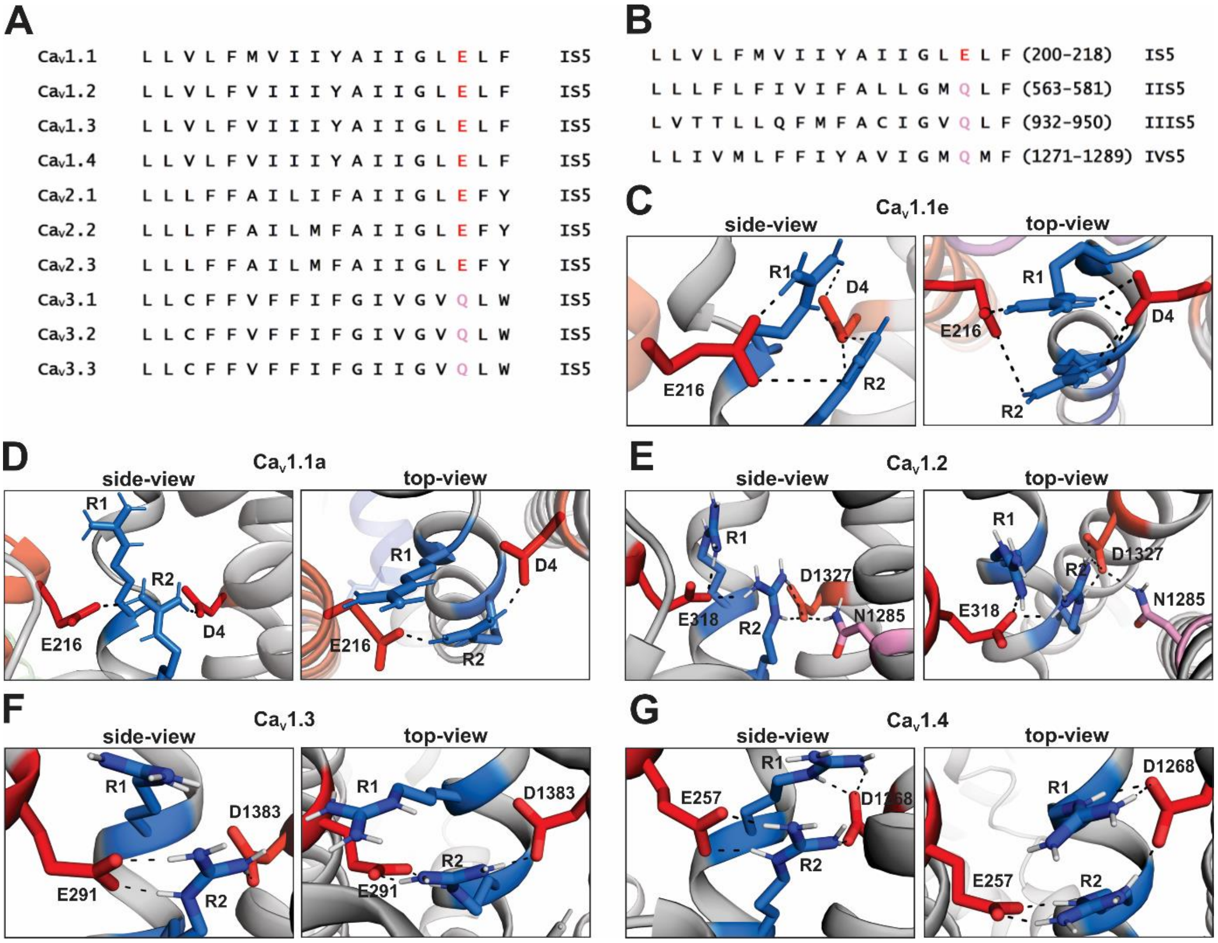

Figure S2. The inter-domain interaction between VSD IV and IS5 is specific for Ca_V1.1 and Ca_V1.2, and in Ca_V1.1 specific for IS5. (**A**) Sequence alignment of the IS5 helix of all ten Ca_V family members (human sequences). Across the high-voltage-activated channels (Ca_V1 and Ca_V2) the sequence identity in IS5 is very high and the negatively charged glutamate (red) corresponding to E216 in Ca_V1.1 is fully conserved. In contrast, the low-voltage-gated Ca_V3 channels contain an uncharged glutamine (pink) in the corresponding position. (**B**) Sequence alignment of the four S5 segments in Ca_V1.1 shows that the glutamate residue E216 (red) is specific for the first repeat (IS5), while the other three repeats (IIS5, IIIS5, IVS5) contain a glutamine (pink) in the corresponding position. Thus, conservation across the Ca_V genes is much higher than in the four repeats of Ca_V1.1, and the inter-domain ion-pair interaction reported here can only occur between VSD IV and PD I. (**C**) In Ca_V1.1e E216 forms ion pairs with both R1 and R2. (**D**) In contrast, in Ca_V1.1a the ion-pair of E216 with R1 is broken. (**E**) Comparable to the situation in Ca_V1.1e, E318 in Ca_V1.2 interacts with both R1 and R2. (**F, G**) In Ca_V1.3 E291 and in Ca_V1.4 E257 interact only with R2 and not with R1, similar to the situation in Ca_V1.1a.

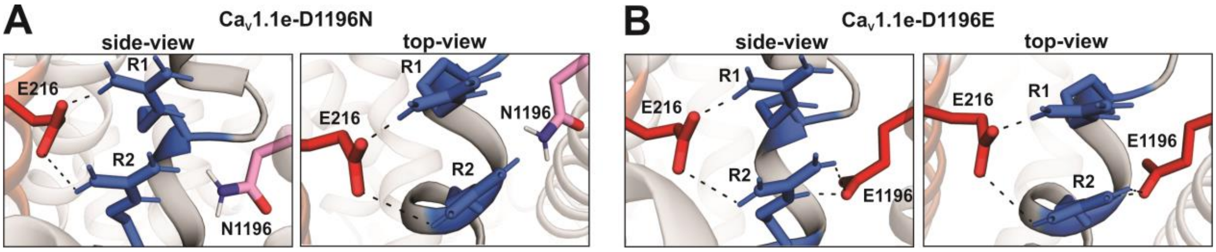

Figure S3. Substitutions of the intra-domain ion-pair partner D1196 eliminate stabilizing interactions with R1 and R2 in Ca_V1.1e. Previously we identified D1196 in IVS3 as a critical intra-domain ion-pair partner stabilizing R1 and R2 of IVS4 in the activated state (15). Substitution of D1196 with N or E caused a substantial right-shift of V½. Homology models of the complete Ca_V1.1e containing these amino acid substitutions shows that in the charge-neutralizing mutation Ca_V1.1e-D1196N the ionic interactions with R1 and R2 are abolished (**A**) and that in the charge-maintaining mutation Ca_V1.1e-D1196E the ionic interactions with R1 is severed as the result of clashes produced by the increased sidechain length (**B**). Importantly, severing these intra-domain interactions does not perturb the inter-domain interactions of R1 and R1 with E216.

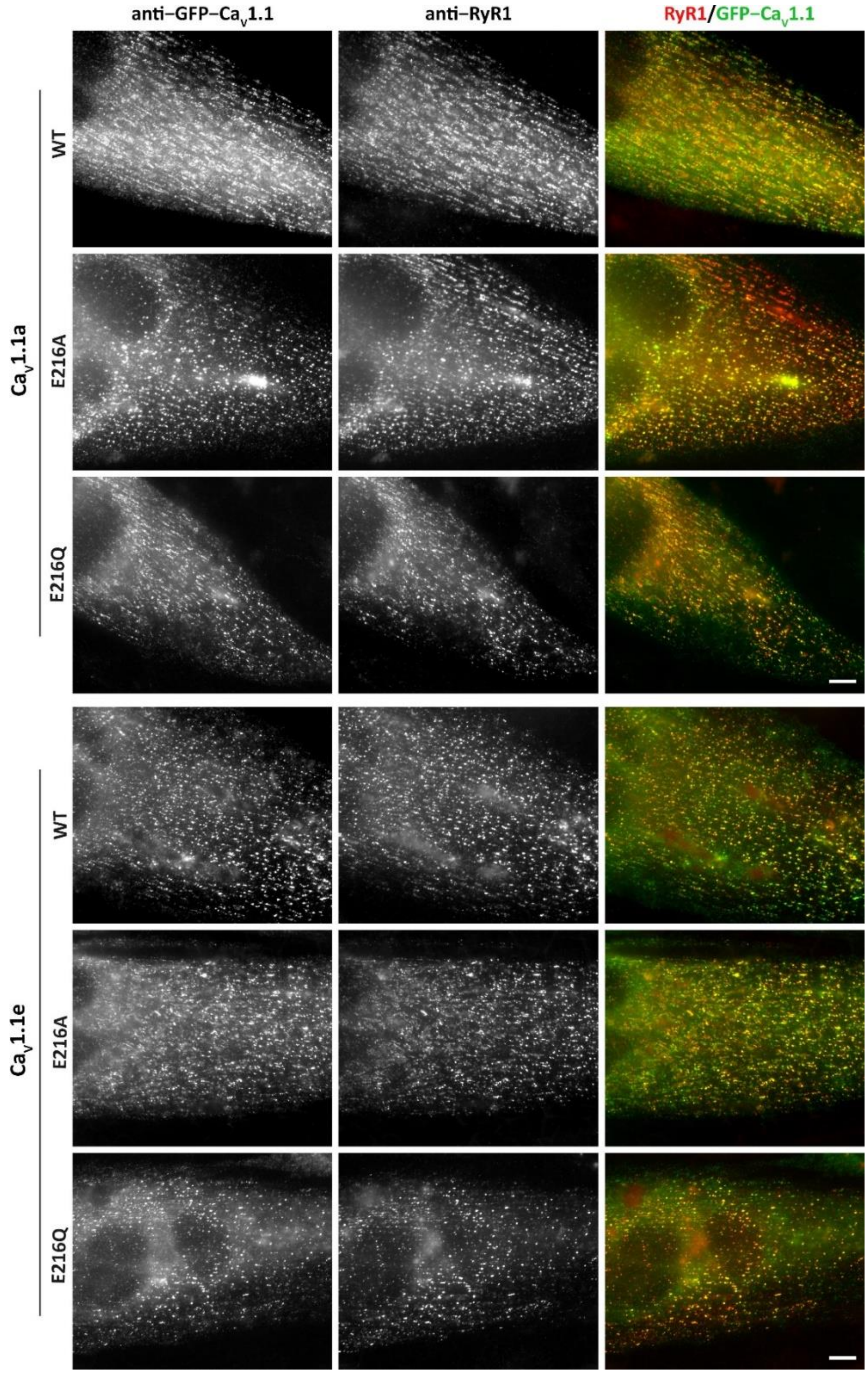

Figure S4: Expression and triad targeting of WT GFP-Ca$_V$1.1a and GFP-Ca$_V$1.1e channels and the E216A and E216Q mutant channels in dysgenic myotubes. Dysgenic (Ca$_V$1.1-null) myotubes were transfected with the indicated GFP-Ca$_V$1.1 constructs and myotubes were fixed and double-immunofluorescence labeled with anti-GFP (to localize the Ca$_V$1.1 construct) and anti-RyR1 (as a triad marker). Colocalization of GFP-Ca$_V$1.1 constructs with RyR1 in clusters indicates its normal incorporation into triadic calcium channel complexes of skeletal myotubes. The E216A and E216Q mutants showed similar expression and distribution as the wildtype GFP-Ca$_V$1.1a and Ca$_V$1.1e. Scale bars, 10 µm.

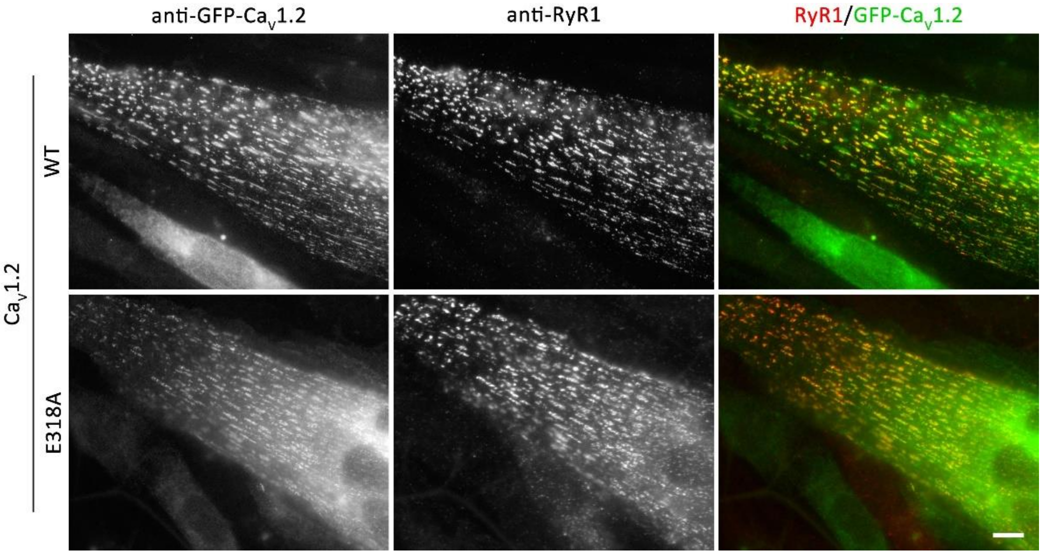

Figure S5. Expression and triad targeting of WT and E318A mutant GFP-Ca_V1.2 channels in dysgenic myotubes. Dysgenic (Ca_V1.1-null) myotubes were transfected with the indicated GFP-Ca_V1.2 constructs and myotubes were fixed and double-immunofluorescence labeled with anti-GFP (to localize the Ca_V1.2 construct) and anti-RyR1 (as a triad marker). Colocalization of GFP-Ca_V1.2 constructs with RyR1 in clusters indicates its normal incorporation into triadic calcium channel complexes of skeletal myotubes. GFP-Ca_V1.2-E318A showed similar expression and distribution as the wildtype GFP-Ca_V1.2. Scale bars, 10 µm.

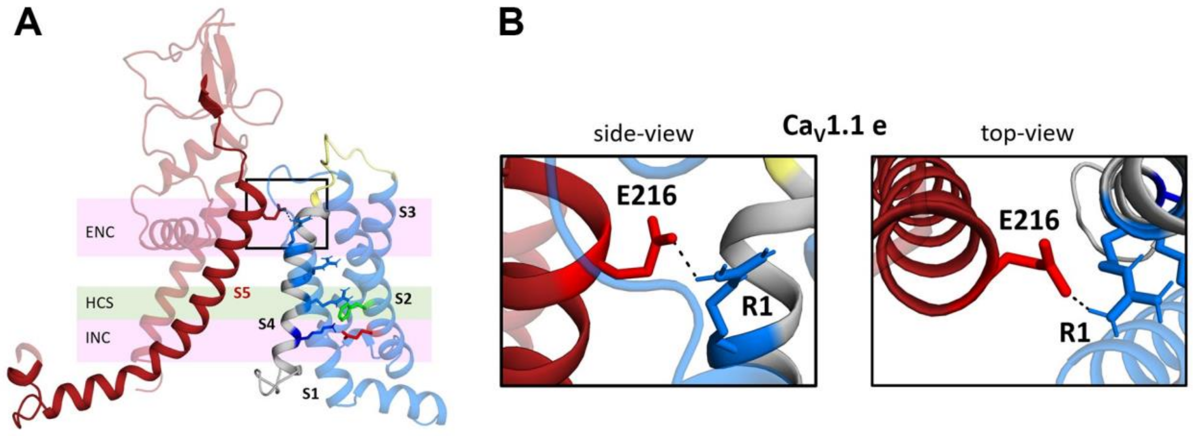

Figure S6. Reduced inter-domain interactions between IVS4 and IS5 in resting state 3 of $Ca_V1.1e$. The recently published resting state 3 structure model of VSD IV (5) was used to characterize the influence of the sliding S4 helix on the inter-domain interaction with IS5. In resting state 3 the S4 helix has moved down one step from the activated state. The resting state 3 model of VSD IV in $Ca_V1.1e$ was obtained by aligning the trans-membrane helices S1-S3 with the VSD IV in the inactivated state and performing an energy minimization in MOE. (**A**) Structure of VSD IV (blue) and the adjacent PD I (red) highlighting the side chains of S4 gating charges (blue) and countercharges (red) of the intracellular negative cluster (INC) and the extracellular negative cluster (ENC), plus the phenylalanine (green) marking the hydrophobic constriction site (HCS). The downward movement of the S4 helix is accompanied by altered interactions formed by the S4 gating charges. In particular, R3 moved into the hydrophobic constriction site and R2 moved out of the ECN. (**B**) Close-up of the remaining inter-domain interaction between R1 with E216 in resting state 3 in $Ca_V1.1e$. Note that the interaction of E216 with R2 is abolished and only one single salt-bridge with R1 remains.

Table S1. Linear interaction energies of the analyzed Ca_V1 isoforms, splice-variants, and mutants.

Ca_V isoforms / mutants	Electrostatic LIE*
$Ca_V1.1e$	-120.4 kcal
$Ca_V1.1a$	<-6 kcal
$Ca_V1.1e/E216A$	-23.8 kcal
$Ca_V1.1e/E216Q$	-47.3 kcal
$Ca_V1.1e/E216A/D1196N$	-12.7 kcal
$Ca_V1.2$	-80.2 kcal
$Ca_V1.3$	<-6 kcal
$Ca_V1.4$	<-8 kcal

* LIE, linear interaction energy
