# Supplementary material for: Ion-pair interactions between voltage-sensing domain IV and pore domain I regulate CaV1.1 gating
Source: Biophys J. 2021 Sep 8;120(20):4429–41. doi: 10.1016/j.bpj.2021.09.004 (PMC8553663; doi:10.1016/j.bpj.2021.09.004)
Supplement: Document S2. Article plus supporting material [file mmc3.pdf]

Biophysical Journal
Article

Ion-pair interactions between voltage-sensing domain IV and pore domain I regulate $Ca_V1.1$ gating

Yousra El Ghaleb,[1] Monica L. Fernández-Quintero,[1,2] Stefania Monteleone,[2,4] Petronel Tuluc,[3] Marta Campiglio,[1] Klaus R. Liedl,[2] and Bernhard E. Flucher[1,*]

[1]Department of Physiology and Medical Physics, Institute of Physiology, Medical University Innsbruck, Innsbruck, Austria; [2]Department of General, Inorganic and Theoretical Chemistry, and Center for Molecular Biosciences Innsbruck; [3]Department of Pharmacology and Toxicology, Institute of Pharmacy and Center for Molecular Biosciences, University of Innsbruck, Innsbruck, Austria; and [4]Evotec (UK) Ltd., Abingdon, Oxfordshire, United Kingdom

ABSTRACT The voltage-gated calcium channel $Ca_V1.1$ belongs to the family of pseudo-heterotetrameric cation channels, which are built of four structurally and functionally distinct voltage-sensing domains (VSDs) arranged around a common channel pore. Upon depolarization, positive gating charges in the S4 helices of each VSD are moved across the membrane electric field, thus generating the conformational change that prompts channel opening. This sliding helix mechanism is aided by the transient formation of ion-pair interactions with countercharges located in the S2 and S3 helices within the VSDs. Recently, we identified a domain-specific ion-pair partner of R1 and R2 in VSD IV of $Ca_V1.1$ that stabilizes the activated state of this VSD and regulates the voltage dependence of current activation in a splicing-dependent manner. Structure modeling of the entire $Ca_V1.1$ in a membrane environment now revealed the participation in this process of an additional putative ion-pair partner (E216) located outside VSD IV, in the pore domain of the first repeat (IS5). This interdomain interaction is specific for $Ca_V1.1$ and $Ca_V1.2$ L-type calcium channels. Moreover, in $Ca_V1.1$ it is sensitive to insertion of the 19 amino acid peptide encoded by exon 29. Whole-cell patch-clamp recordings in dysgenic myotubes reconstituted with wild-type or E216 mutants of GFP-$Ca_V1.1$e (lacking exon 29) showed that charge neutralization (E216Q) or removal of the side chain (E216A) significantly shifted the voltage dependence of activation ($V_{1/2}$) to more positive potentials, suggesting that E216 stabilizes the activated state. Insertion of exon 29 in the GFP-$Ca_V1.1$a splice variant strongly reduced the ionic interactions with R1 and R2 and caused a substantial right shift of $V_{1/2}$, whereas no further shift of $V_{1/2}$ was observed on substitution of E216 with A or Q. Together with our previous findings, these results demonstrate that inter- and intradomain ion-pair interactions cooperate in the molecular mechanism regulating VSD function and channel gating in $Ca_V1.1$.

SIGNIFICANCE Voltage-gated calcium channels (Ca_Vs) regulate the excitability and synaptic transmission in nerve cells and contraction of skeletal and heart muscles. How cation channels sense electrical signals and transduce them into channel openings with characteristic gating properties is still incompletely understood. The four voltage-sensing domains (VSDs) of Ca_V channels represent independent functional units with a common building plan but distinct biophysical properties. Upon depolarization, movement of the positively charged S4 helix through the membrane electrical field is aided by transient interactions with countercharges within the VSD. Here, structure modeling and mutagenesis experiments revealed the participation of an ion-pair partner outside the VSD in regulation of the voltage-sensing process of VSD IV in $Ca_V1.1$ and its modulation by alternative splicing.

INTRODUCTION

Activation of voltage-gated cation channels is controlled by a set of four voltage-sensing domains (VSDs), symmetrically arranged around a common ion conduction pore (Fig. 1, *A and B*) (1). VSDs are highly conserved functional modules composed of four membrane-spanning helices (S1–S4) (2). Upon membrane depolarization, the positively charged S4

Submitted April 15, 2021, and accepted for publication September 2, 2021.

*Correspondence: bernhard.e.flucher@i-med.ac.at

Yousra El Ghaleb and Monica L. Fernández-Quintero contributed equally to this work.

Stefania Monteleone's present address is Evotec (UK) Ltd., Abingdon, United Kingdom.

Editor: Lucie Delemotte.

https://doi.org/10.1016/j.bpj.2021.09.004

© 2021 Biophysical Society.

This is an open access article under the CC BY license (http://creativecommons.org/licenses/by/4.0/).

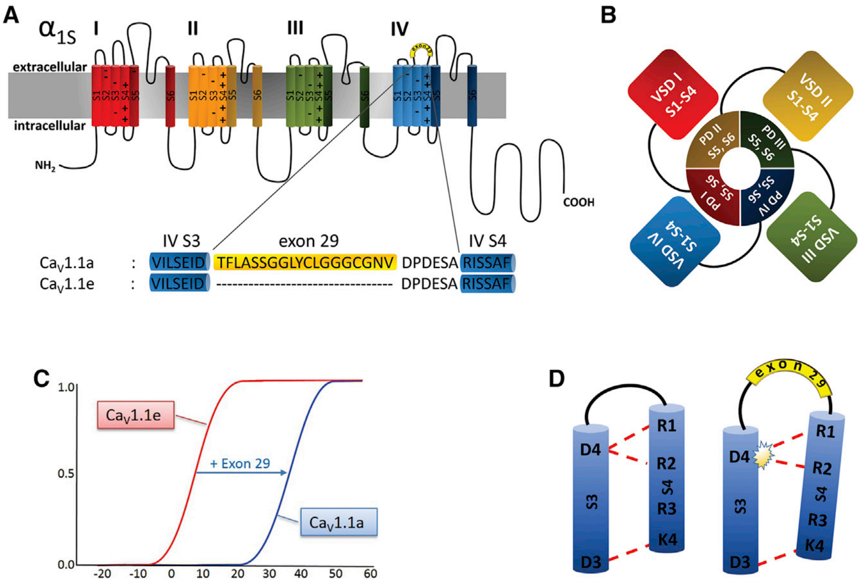

FIGURE 1 Domain structure of $Ca_V1.1$ and proposed effect of alternative splicing of exon 29 on ion-pair formation in VSD IV. (*A*) Domain structure of $Ca_V1.1$ comprising four homologous repeats (I, II, III, and IV) with six transmembrane helices (S1–S6) each. The alternatively spliced exon 29 (*yellow*) is inserted in the IVS3-S4 linker of $Ca_V1.1a$, but lacking in $Ca_V1.1e$. (*B*) In the domain-swapped arrangement of Ca_V channels, the VSD (S1–S4) of one repeat is positioned next to the PD of the adjacent repeat. (*C*) Drawing of the conductance to voltage curves indicates the 30 mV right shift of the voltage dependence of activation induced by the insertion of exon 29. (*D*) Conceptual model depicting how insertion of exon 29 between IVS3 and IVS4 disrupts ion-pair formation of the gating charges R1 and R2 with the countercharge D1196 (D4) (3,4). To see this figure in color, go online.

helix is moved outward relative to S1–S3, and the ensuing conformational change is transmitted to opening of the channel gate via a cytoplasmic S4-S5 linker. In this process, evenly spaced arginines and lysines in S4 (termed gating charges, R1, R2, …) are sequentially translocated across the membrane electric field, which is highly focused at the hydrophobic constriction site in the center of the VSD (5). The energetically unfavorable movement of the positively charged amino acids through the hydrophobic environment is supported by the transient formation of ion pairs between the gating charges and negative countercharges in the S2 and S3 helices (called charge-transfer center or inner negative cluster). Additional ion pairs between the gating charges and countercharges in an extracellular negative cluster (ENC) help stabilize VSDs in successive resting and activated states and thus contribute to shaping the characteristic gating properties of different channels (6,7).

Potassium channels and prokaryotic sodium channels are built of four subunits, each comprising a single VSD and part of the pore (S5-S6; pore domain, PD) (8). In eukaryotic sodium (Na_V) and calcium (Ca_V) channels, the pore-forming subunit is the product of a single gene, and its four VSDs are structurally and functionally distinct (9,10). Specifically, in the skeletal muscle calcium channel $Ca_V1.1$, the four VSDs differ in the number and type of gating charges as well as in the number, type, and position of their countercharges (11). These distinct structural features determine the characteristic aspects of $Ca_V1.1$'s channel gating properties, such as the extremely slow activation kinetics, which is controlled by VSD I, and the very right-shifted voltage dependence of activation, which is controlled by VSD IV (3,12).

In addition to their channel-related function, in skeletal muscle the VSDs of $Ca_V1.1$ activate calcium release from the sarcoplasmic reticulum. In this process, called excitation-contraction (EC) coupling, the conformational change of one or more VSDs is allosterically transmitted to the opening of the sarcoplasmic reticulum calcium release channel, the type 1 ryanodine receptor (13). Importantly, both the kinetics and voltage dependence of EC coupling differ from those of the calcium currents; depolarization-induced calcium release is faster and occurs at ~30 mV less depolarized potentials compared to activation of currents in the adult $Ca_V1.1a$ isoform (12,14,15). These observations support the hypothesis that less than all four VSDs are necessary for activation of EC coupling and that VSD I and VSD IV are probably not involved in this chief physiologically function of $Ca_V1.1$ (3). This notion is further substantiated by the finding that inclusion of the alternatively spliced exon 29 in the IVS3-S4 linker markedly right shifts the voltage dependence of current activation without affecting the voltage dependence of EC coupling (Fig. 1 *C*; (15)).

In our ongoing efforts to understand the molecular mechanisms underlying the specific properties of the $Ca_V1.1$ VSDs and their differential contribution to regulating L-type calcium currents and EC coupling, we recently identified an ion-pair partner of the outer gating charges (R1/R2) of VSD IV that is of critical importance for the regulation of the gating properties by alternative splicing (4). Charge-neutralizing mutations of D1196 (D4) at the extracellular end of IVS3 or its putative ion-pair partners R1 and R2 in IVS4 all right-shifted $V_{1/2}$ and decreased the current density similarly to the insertion of the 19 amino acid exon 29 in the loop connecting IVS3 and IVS4. This finding gave rise to a mechanistic model according to which the formation of ion pairs between R1/R2 and D1196 stabilize the VSD IV in the activated state, causing left-shifted, high-amplitude currents,

and this molecular interaction is disrupted by the insertion of exon 29, causing the prominent right shift and attenuation of the currents in the adult $Ca_V1.1a$ splice variant (Fig. 1 *D*).

Here, we revisited this question and examined this mechanism in advanced structure models of the complete $Ca_V1.1$, with and without exon 29, and discovered an additional ion-pair partner of R1/R2 of VSD IV. This putative interaction partner (E216) previously had been missed in the structure models of the isolated VSD IV (4) because it resides outside this VSD in the PD of the first repeat (IS5). Mutagenesis of E216 and electrophysiological analysis show that its mutation causes a right shift of $V_{1/2}$ similar to that seen previously on mutation of D1196, R1, or R2. This effect is observed only in the $Ca_V1.1e$ splice variant (lacking exon 29) and not in $Ca_V1.1a$ (containing exon 29). Our data suggest that E216 in IS5 and D1196 in IVS3 serve as ion-pair partners of R1/R2 in IVS4, together stabilizing VSD IV in the activated state, and that upon insertion of exon 29, this interdomain interaction becomes disrupted, resulting in the right-shifted and low-amplitude L-type calcium currents characteristic of mature skeletal muscle cells.

MATERIALS AND METHODS

Structure modeling

The structures of both splice variants of the human α_1-subunit ($Ca_V1.1e$ and $Ca_V1.1a$) were modeled based on the rabbit cryo-electron microscopy (EM) structure of $Ca_V1.1$ in the inactivated state, with voltage sensors in the "up" conformation and a closed intracellular gate (Protein Data Bank: 5GJV) (11).

Homology modeling has been performed using Rosetta and MOE (Molecular Operating Environment, version 2018.08; Molecular Computing Group, Montreal, Canada). Additionally, ab initio Rosetta modeling (16) was used to generate structures for loops that were not resolved in the original Cav1.1 α_1-subunit template (17). The structures for the E216A/Q mutants were derived from both wild-type (WT) splice variant models by replacing the mutated residue and carrying out a local energy minimization using MOE. The C-terminal and N-terminal parts of each domain were capped with acetylamide and N-methylamide to avoid perturbations by free charged functional groups. The structure model was embedded in a plasma membrane consisting of 1-palmitoyl-2-oleoyl-*sn*-glycero-3-phosphocholine and cholesterol in a 3:1 ratio, using the CHARMM graphical user interface (GUI) Membrane Builder (18,19). Water molecules and 0.15 M KCl were included in the simulation box. Energy minimizations of $Ca_V1.1e$ and $Ca_V1.1a$ WT and mutant structures in the membrane environment were performed. The topology was generated with the LEaP tool of the AmberTools18 (20), using force fields for proteins and lipids, ff14SBonlysc and Lipid14 (21), respectively. The structure models were heated from 0 to 300 K in two steps, keeping the lipids fixed, and then equilibrated over 1 ns. Then, molecular dynamics (MD) simulations were performed for 800 ns, with time steps of 2 fs, at 300 K and in anisotropic pressure scaling conditions (22). Van der Waals and short-range electrostatic interactions were cut off at 10 Å, and long-range electrostatics were calculated by the particle mesh Ewald method. MOE and PyMOL were used to visualize the key interactions and point out differences in structure models (The PyMOL Molecular Graphics System, Version 2.0; Schrödinger, LLC.). Additionally, homology models for the other Ca_V1 isoforms $Ca_V1.2$, $Ca_V1.3$, and $Ca_V1.4$ were generated based on the rabbit cryo-EM structure of $Ca_V1.1$ and relaxed and equilibrated in the membrane environment. This allowed us to identify interactions specific for certain Ca_V1 isoforms. The high sequence conservation among the α_1-subunit in Ca_V1 isoforms allowed a reliable structure model prediction. To capture functionally relevant side-chain rearrangements and to quantify the lifetime and stability of the interdomain interactions, we performed 250 ns of all-atom MD simulations of $Ca_V1.2$ and compared them to the first 250 ns of the $Ca_V1.1a$ and $Ca_V1.1e$ simulations.

Linear interaction energies were calculated to estimate the strength of the electrostatic interactions using the tool LIE (linear interaction energy) implemented in cpptraj.

Plasmids

Cloning procedures for GFP-$Ca_V1.1a$ WT, GFP-$Ca_V1.1e$ WT, and GFP-$Ca_V1.2$ WT were previously described (15,23).

To generate GFP-$Ca_V1.1a$-E216Q and GFP-$Ca_V1.1e$-E216Q, amino acid (aa) E216 was mutated using splicing by overlap extension polymerase chain reaction (SOE-PCR). Briefly, nucleotides (nt) 1–1113 of $Ca_V1.1a$ (nt 226–1338 of CACNA1S, National Center for Biotechnology Information (NCBI) reference sequence NM_001101720.1) were PCR amplified with overlapping primers introducing the point mutation G > C at position nt 646 (nt 871 of NM_001101720.1) for the E216A in separate PCR reactions using GFP-$Ca_V1.1a$-WT as template. The two separate PCR products were then used as templates for a final PCR reaction with flanking primers to connect the nucleotide sequences. These fragments were then SalI/*Eco*RI digested and cloned into the respective sites of GFP-$Ca_V1.1a$ or GFP-$Ca_V1.1e$.

To generate GFP-$Ca_V1.1a$-E216A, aa E216 was neutralized by SOE-PCR. Briefly, nt 1–1113 of $Ca_V1.1a$ (nt 226–1338 of NM_001101720.1) were PCR amplified with overlapping primers introducing the point mutation A > C at position nt 647 (nt 872 of NM_001101720.1) in separate PCR reactions using GFP-$Ca_V1.1a$-WT as template. The two separate PCR products were then used as templates for a final PCR reaction with flanking primers to connect the nucleotide sequences. These fragments were then SalI/*Eco*RI digested and cloned into the respective sites of GFP-$Ca_V1.1a$.

To generate GFP-$Ca_V1.1e$-E216A, nt 2654–4430 of the $Ca_V1.1e$ coding sequence (nt 2879–4712 of CACNA1S NCBI reference sequence NM_001101720.1) were isolated from GFP-$Ca_V1.1e$-WT by digestion with XhoI and BglII and inserted in the corresponding sites of GFP-$Ca_V1.1a$-E216A, yielding GFP-$Ca_V1.1e$-E216A.

To generate GFP-$Ca_V1.2$-E318A, aa E318 was mutated by SOE-PCR. Briefly, nt 1–1368 of $Ca_V1.2$ (nt 192–1559 of CACNA1C NCBI reference sequence X15539) were PCR amplified with overlapping primers introducing the point mutation A > C at position nt 953 (nt 1144 of X15539) in separate PCR reactions using GFP-$Ca_V1.2$ WT as a template. The two separate PCR products of each construct were then used as templates for a final PCR reaction with flanking primers to connect the nucleotide sequences. These fragments were then SalI/BamHI digested and cloned into the respective sites of GFP-$Ca_V1.2$.

All newly generated plasmids were verified by sequencing (Eurofins Genomics, Ebersberg, Germany).

Cell culture and transfection

Myoblasts of the dysgenic (mdg/mdg) cell line GLT were cultured as previously described in Powell et al. (24). Briefly, cells were plated on 35 mm culture dishes and transfected with 0.5 μg of the desired Ca_V1 subunit 4 days after plating using FuGENE-HD transfection reagent (Promega, Madison, WI). Transfected myotubes were analyzed by electrophysiology after 7–8 days in culture or fixed for immunolabeling after 9–10 days in culture. The auxiliary calcium channel subunits $\alpha_2\delta$-1, β_{1a}, and γ_1, along with the STAC3 protein and ryanodine receptor, are endogenously expressed in GLT myotubes, enabling functional membrane incorporation of the channel constructs in the triad junction.

Immunofluorescence

Paraformaldehyde-fixed cultures were immunolabeled as previously described (25) with rabbit polyclonal anti-GFP (1:10,000; Invitrogen, Thermo Fisher Scientific, Waltham, MA) and mouse monoclonal anti-ryanodine receptor (34-C; 1:500; Invitrogen, Thermo Fisher Scientific) and fluorescently labeled with goat anti-rabbit Alexa-488 and secondary goat anti-mouse Alexa-594 (1:4000), respectively. Thus, the anti-GFP label and the intrinsic GFP signal were both recorded in the green channel. Samples were observed using a 60×, 1.42 NA objective with a BX53 Olympus microscope, and 14-bit images were captured with a cooled charge-coupled device camera (XM10; Olympus, Tokyo, Japan) and CellSens Dimension image-processing software (Olympus). Image composites were arranged in Adobe Photoshop CS6 (Adobe Systems), and linear adjustments were performed to correct black level and contrast.

Electrophysiology

Calcium currents were recorded with the whole-cell patch-clamp technique in voltage-clamp mode using an Axopatch 200A amplifier (Axon Instruments, Molecular Devices, San Jose, CA). Patch pipettes (borosilicate glass; Science Products, Hofheim, Germany) had resistances between 1.5 and 3.5 MΩ when filled with 145 mM Cs-aspartate, 2 mM $MgCl_2$, 10 mM HEPES, 0.1 mM Cs-EGTA, and 2 mM Mg-ATP (pH 7.4 with CsOH). The extracellular bath solution contained 7.5 mM $CaCl_2$ (10 mM $CaCl_2$ for $Ca_V1.2$ and E216A/D1196N data sets), 145 mM tetraethylammonium chloride, and 10 mM HEPES (pH 7.4 with tetra-ethylammonium hydroxide). Data acquisition and command potentials were controlled by pCLAMP software (Clampex version 10.2; Axon Instruments); analysis was performed using Clampfit 10.7 (Axon Instruments) and SigmaPlot 12.0 (SPSS Science) software. The current-voltage dependence was fitted according to

$$I = G_{max} \times (V - V_{rev}) / \left(1 + \exp\left(-\left(V - V_{1/2}\right)/k\right)\right),$$

where G_{max} is the maximal conductance of the L-type calcium currents, V_{rev} is the extrapolated reversal potential of the calcium current, $V_{1/2}$ is the potential for half maximal conductance, and k is the slope. The conductance was calculated using $G = (-I \times 1000)/(Vrev - V)$, and its voltage dependence was fitted according to a Boltzmann distribution:

$$G = G_{max} / \left(1 + \exp\left(-\left(V - V_{1/2}\right)/k\right)\right).$$

Statistics

All eight experimental groups were analyzed in transiently transfected cells each from three to eight independent cell passages. The E216Q and E216A variants of $Ca_V1.1a$ and $Ca_V1.1e$ were recorded in parallel with the WT channel in cells of the same passage. Also, the E318A variant of $Ca_V1.2$ was always recorded in parallel with the WT $Ca_V1.2$ in cells of the same passage. The means, standard errors, and p-values were calculated using Student's t-test, two-tailed, or in the case of $Ca_V1.1e$-E216A/D1196N by using an analysis of variance (ANOVA) with Tukey's posthoc test, with significance criteria $^*p < 0.05$, $^{**}p < 0.01$, and $^{***}p < 0.001$).

RESULTS

Structure prediction of splice-dependent interactions between IVS4 gating charges and an ion-pair partner in IS5

Structure models of the human $Ca_V1.1a$ and $Ca_V1.1e$ splice variants (Fig. 1), with the VSDs in the activated up-state, were generated based on the cryo-EM structure of $Ca_V1.1$ (11) (Fig. 2 *A*). At a resolution of 3.6 Å, the cryo-EM structure falls short of showing the details of atomic orientation necessary to describe the molecular interactions formed by the gating charges. To achieve this, we first included nonresolved intracellular and extracellular loops in our model using *Rosetta* modeling. The structure of the extracellular loop connecting S3 and S4 in the fourth VSD was also modeled without the 19 amino acids encoded by the alternatively spliced exon 29, to generate the structure of the embryonic splice variant $Ca_V1.1e$. We then relaxed and equilibrated the modeled structures of both splice variants in the membrane environment for 800 ns to capture side-chain rearrangements and interactions. In contrast to big domain movements, like the sliding of the S4 helix, side-chain orientations in a specific state can be sampled in the femto- to nanosecond timescale (26). Simulation of side-chain dynamics allows us to assess the importance of specific H-bonds and ionic interactions. Specifically, the presence and duration of a given interaction over the considered simulation time, provides a reliable prediction of its relevance and functional role. Thus, our conformational ensembles helped identifying potentially functionally relevant interactions of the gating charges, which remained unnoticed before when only a single static structure had been considered (4).

We focused our attention on ionic interactions of gating charges in VSD IV because we already had extensive structural and functional data on this VSD from previous structure-function studies (4,15,27). These studies suggested a regulatory mechanism by which, in $Ca_V1.1e$, an interaction with a countercharge in IVS3 (D1196, or D4) stabilizes R1 and R2 of IVS4 in the activated state and this interaction is disrupted by the insertion of exon 29 into the IVS3-S4 linker of $Ca_V1.1a$ (4). As predicted, the current structure model of $Ca_V1.1e$ showed that in the activated state, D1196 forms two strong interactions each with the side chains of R1 and R2. And, also as proposed previously, in $Ca_V1.1a$ the interactions with R1 were abolished and those with R2 reduced to a single ionic interaction (Fig. 2, *B and C*). Unexpectedly, the structures revealed the participation of an additional ion-pair partner of R1 and R2. In $Ca_V1.1e$ E216 forms ionic interactions with the side chains of both outer gating charges R1 and R2. Again, on insertion of exon 29 (i.e., in $Ca_V1.1a$), these ion bonds were reduced to a single salt bridge with R2, suggesting a function of E216 in the splicing-dependent stabilization of $Ca_V1.1e$ VSD IV in the activated state. Importantly, E216 resides outside of VSD IV at the extracellular end of the S5 helix in the PD of repeat I. In the domain-swapped channel arrangement, the PD of repeat I is located immediately adjacent to the VSD of repeat IV (Video S1). The observed loss of the ionic interactions with R1 as a consequence of the insertion of exon 29 (i.e., in $Ca_V1.1a$) can be mechanistically explained by the altered orientations of the S3, S4, and S5 helices. Alternative splicing of exon 29 results in a displacement of the Cα-atoms of R1, E216, and

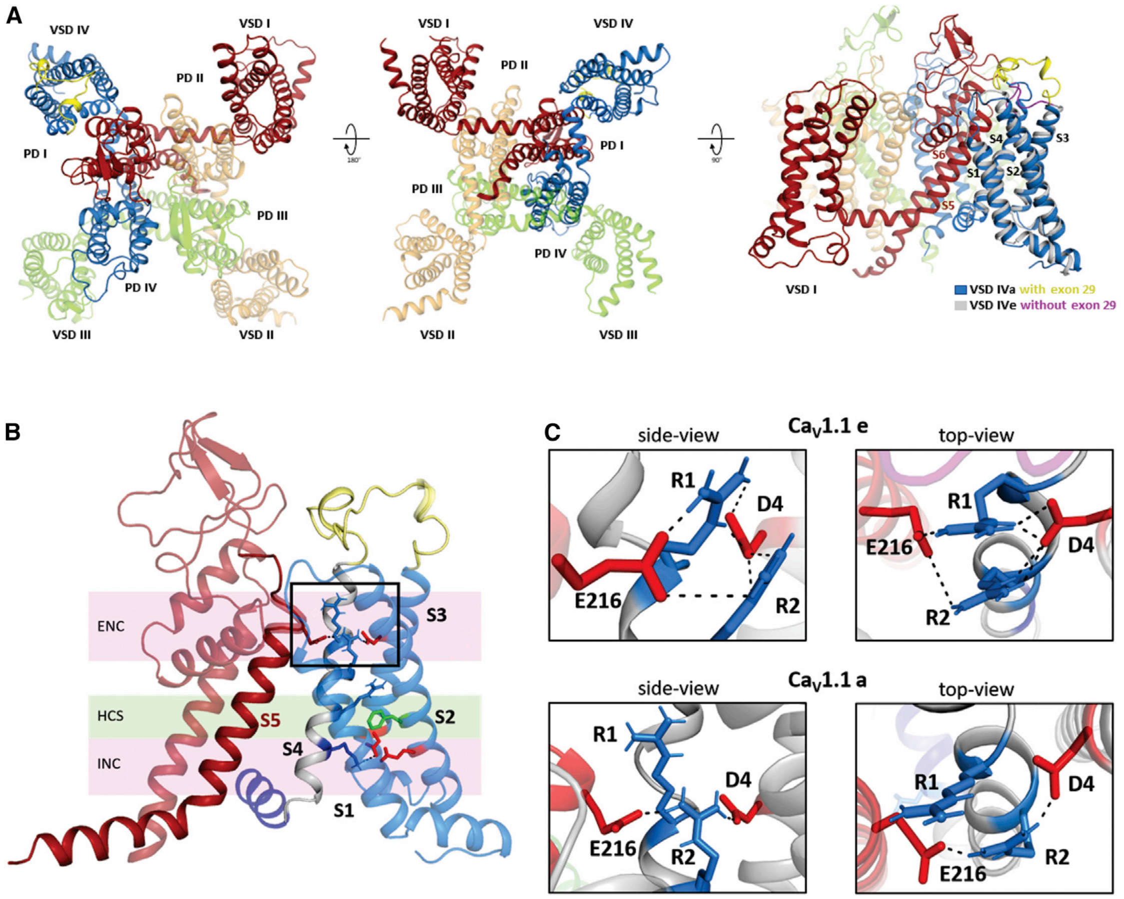

FIGURE 2 Structure models predict ionic interactions of R1/R2 gating charges in IVS4 stabilizing the activated state of $Ca_V1.1e$, but not of $Ca_V1.1a$. (*A*) Structure model of $Ca_V1.1$ with the four repeats shown in distinct colors. Note that the VSDs are positioned adjacent to the PD of the next repeat in a clockwise orientation. VSD IV is shown with and without exon 29 (*blue*/*yellow* or *gray*/*pink*, respectively). (*B*) Structure of VSD IV (*blue*) and the adjacent PD I (*red*) highlighting the side chains of S4 gating charges (*blue*) and countercharges (*red*) of the intracellular negative cluster (INC) and the extracellular negative cluster (ENC), plus the phenylalanine (*green*) marking the hydrophobic constriction site (HCS). (*C*) Close-ups of the contact interface between PD I and VSD IV (marked by the *frame* in *B*), showing the hydrogen bonds (*dashed lines*) between R1, R2, and ion-pair partners D1196 (D4) in IVS3 and E216 in the PD of IS5. Note that in $Ca_V1.1a$ (including exon 29), the ionic interactions are absent (R1) or diminished (R2). To see this figure in color, go online.

D1196 of ~4–7 Å and changes of the relative distances between them of ~1 Å (Fig. S1).

To examine whether this interdomain ion-pair interaction is specific to the fourth VSD of $Ca_V1.1$ or a regular feature in the structure of all its VSDs, we inspected the corresponding PD positions in VSDs II, III, and IV for the existence of analogous residues that might serve as ion-pair partners for the respective adjacent VSD. Although the sequence at the outer end of S5 is highly conserved in all four repeats of Ca_V channels (Fig. S2 *A*), the three other PDs (II, III, and IV) all contain an uncharged glutamine in the corresponding position (Fig. S2 *B*), suggesting that this putative countercharge is unique for IS5.

E216 mutations in $Ca_V1.1e$ destabilize the ion-pair interactions with R1 and R2 and right shift the voltage dependence of current activation

Previously, we demonstrated that neutralizing the countercharge D1196 (D1196N) or changing the length of its side chain (D1196E) both right-shifted $V_{1/2}$ of activation similarly as mutating the IVS4 gating charges R1 and R2 themselves (4). This led to the conclusion that in $Ca_V1.1e$ R1 and R2 of VSD IV interact with D1196 and that this interaction stabilizes the channel in the activated state. Consistent with this notion, homology modeling the structures of $Ca_V1.1e$ containing these amino acid substitutions showed that in $Ca_V1.1e$-D1196N, the ionic interactions with R1 and R2 are abolished (Fig. S3 *A*), and in $Ca_V1.1e$-D1196E they are severed as the result of multiple clashes produced by the increased side-chain length (Fig. S3 *B*). In addition to these intra-VSD interactions, the structure models of the complete channel now revealed the participation in this process of an R1/R2 interaction partner (E216) outside of VSD IV (Fig. 2, *B and C*). If the predicted interdomain interactions of E216 with R1 and R2 are functionally relevant and also contribute to stabilizing VSD IV in the activated state, neutralizing or diminishing its side chain should affect the voltage dependence of activation, similarly to what has

been observed previously with the corresponding substitutions of D1196 (4). To test this hypothesis, we generated two constructs of the GFP-tagged rabbit $Ca_V1.1e$ with substitutions of E216: one in which the charged side chain has been replaced by alanine (E216A) and a second, more conservative substitution with glutamine (E216Q), which removed the charge but otherwise maintained the chemical nature and length of the side chain. Notably, the latter substitution resembles the amino acid found in the corresponding positions of VSDs II, III, and IV (Fig. S2 *B*).

Homology modeling of the $Ca_V1.1e$ channel with the E216A substitution demonstrated that the interactions with the outer gating charges were completely severed (Fig. 3 *A*). In contrast, in E216Q the glutamine in position 216 still interacted with both outer gating charges R1 and R2 of VSD IV, albeit considerably weaker than the glutamate in the wild-type channel. Because of the charge neutralization, the ionic bonds were replaced by weaker H-bonds (Fig. 3 *B*). We expressed these mutated constructs and the wild-type control in dysgenic ($Ca_V1.1$-null) myotubes for electrophysiological analysis of their current properties in the native muscle cell environment. Regular expression and targeting of the recombinant GFP-$Ca_V1.1e$ constructs into skeletal muscle triads was confirmed with immunofluorescence labeling (Fig. S4). The GFP fluorescence guided the selection of transfected myotubes for patch-clamp analysis.

Consistent with our hypothesis, the alanine substitution (E216A) in GFP-$Ca_V1.1e$ resulted in calcium currents with substantially right-shifted voltage dependence of activation compared to wild-type control (GFP-$Ca_V1.1e$: 6.0 ± 0.7 mV; GFP-$Ca_V1.1e$-E216A: 21.7 ± 1.6 mV; $p < 0.0001$) (Fig. 3, *C–F*). Current density was only slightly reduced; however, kinetics of activation was slowed, and the fractional inactivation at the end of the 500 ms test pulse was reduced to about half of control values (Table 1). Also, charge neutralization (E216Q) in GFP-$Ca_V1.1e$ resulted in a highly significant, albeit smaller, right shift of voltage dependence of activation compared to wild-type controls (GFP-$Ca_V1.1e$: 10.0 ± 1.2 mV; GFP-$Ca_V1.1e$-E216A: 16.8 ± 1.2 mV; $p < 0.001$) (Fig. 3, *G–J*). Again, current density was somewhat reduced (Table 1). The right-shifted voltage dependence of activation in both mutants indicates the involvement of E216 in the regulation of channel gating, specifically in the stabilization of the activated state. The different extent of this effect in

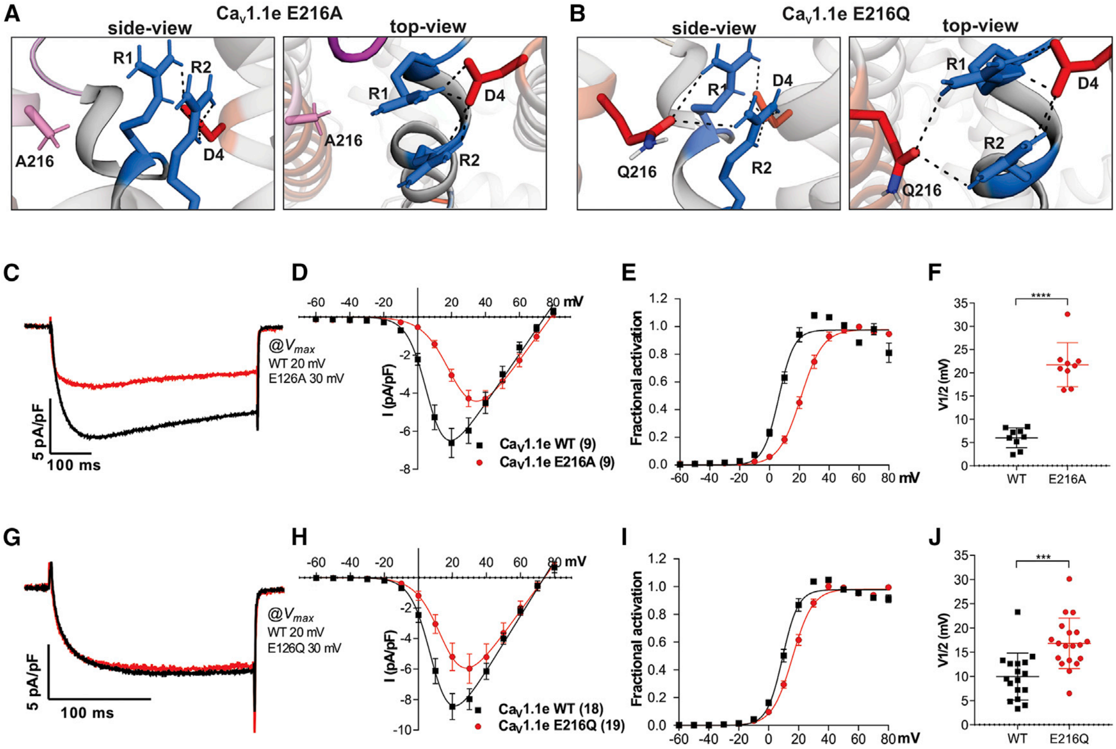

FIGURE 3 Mutations of E216 in $Ca_V1.1e$ (lacking exon 29) alter the interactions with R1/R2 and channel gating properties. (*A* and *B*) Structures of the PD I – VSD IV interface of $Ca_V1.1e$ substitutions E216A (*A*) and E216Q (*B*), indicating the ion bonds and H-bonds (*dashed lines*) between the gating charges R1/R2 (*blue*), the countercharge D1196 (*red*), and the two substitutions for E216 (*pink*/*red*). Note that the substitutions of E216 perturb its interactions with R1 and R2 without affecting their interactions with D1196 (D4) (c.f. wild-type in Fig. 2 *C*). (*C–F*) Representative example traces (*C*), current-voltage (I-V) curves (*D*), and conductance-voltage (G-V) curves (*E*) of calcium currents recorded from myotubes expressing wild-type GFP-$Ca_V1.1e$ (*black*) or mutant GFP-$Ca_V1.1e$-E216A (*red*). (*F*) The E216A substitution shifts $V_{1/2}$ in the depolarizing direction by 15.7 mV (N = 9; $p < 0.0001$). (*G–J*) Representative example traces (*G*), I-V curves (*H*), and G-V curves (*I*) of calcium currents recorded from myotubes expressing wild-type GFP-$Ca_V1.1e$ (*black*) or mutant GFP-$Ca_V1.1e$-E216Q (*red*). (*J*) The E216Q substitution shifts $V_{1/2}$ in the depolarizing direction by 6.8 mV (N = 17–19; $p < 0.001$). P-values were calculated using a student t-test, error bars indicate standard error. For further current parameters, see Table 1. To see this figure in color, go online.

TABLE 1 Current properties of $Ca_V1.1e$, $Ca_V1.1a$, and their mutants E216A, E216Q, and E216A/D1196N and of $Ca_V1.2$ and its mutant E318A

Parameters	$Ca_V1.1e$ WT	$Ca_V1.1e$ E216A	*p*-Value	$Ca_V1.1e$ WT	$Ca_V1.1e$ E216Q	*p*-Value
I_{peak} (pA/pF)	−6.6 ± 0.8	−4.5 ± 0.4	0.03*	−8.8 ± 0.8	−6.0 ± 1.0	0.04*
G_{max} (nS/nF)	121.4 ± 11.9	123.0 ± 12.5	0.93	159.4 ± 17.0	150.4 ± 19.4	0.7
$V_{1/2}$ (mV)	6.0 ± 0.7	21.7 ± 1.6	****	10.0 ± 1.2	16.8 ± 1.2	***
k_{act} (mV)	5.0 ± 0.3	7.5 ± 0.6	***	5.1 ± 0.2	6.9 ± 0.5	0.003**
V_{rev} (mV)	74.4 ± 1.2	78.1 ± 1.3	0.05	74.7 ± 1.4	72.9 ± 1.4	0.47
Time to peak (ms)	75.6 ± 7.9	137.7 ± 25.7	0.03*	96.6 ± 12.1	82.0 ± 11.2	0.38
R_{500} (%)	30.0 ± 1.7	14.7 ± 2.6	****	N/A	N/A	N/A
n (*n Ipeak*)	9 (9)	9 (9)	N/A	17 (18)	19 (19)	N/A
Parameters	$Ca_V1.1a$ WT	$Ca_V1.1a$ E216A	*p*-Value	$Ca_V1.1a$ WT	$Ca_V1.1a$ E216Q	*p*-Value
I_{peak} (pA/pF)	−2.0 ± 0.3	−1.7 ± 0.3	0.32	−3.4 ± 0.7	−1.9 ± 0.3	0.06
G_{max} (nS/nF)	95.1 ± 15.1	70.8 ± 19.3	0.33	165.8 ± 35.9	120.8 ± 26.2	0.32
$V_{1/2}$ (mV)	33.6 ± 3.7	30.0 ± 3.3	0.49	34.7 ± 2.8	37.2 ± 2.7	0.54
k_{act} (mV)	12.1 ± 1.3	13.3 ± 0.5	0.41	11.4 ± 0.9	10.6 ± 1.1	0.62
V_{rev} (mV)	77.7 ± 1.2	79.4 ± 2.0	0.44	72.8 ± 2.1	69.8 ± 1.4	0.23
Time to peak (ms)	208.8 ± 38.9	253.8 ± 55.9	0.51	129.6 ± 30.4	119.2 ± 13.9	0.73
R_{500} (%)	13.0 ± 3.4	13.7 ± 4.8	0.91	N/A	N/A	N/A
n (*n Ipeak*)	8 (10)	7 (8)	N/A	7 (7)	10 (10)	N/A
Parameters	$Ca_V1.1e$ WT	$Ca_V1.1a$ WT	*p*-Value (vs. $Ca_V1.1e$ WT)	$Ca_V1.1e$ E216A/D1196N	*p*-Value (vs. $Ca_V1.1e$ WT)	
I_{peak} (pA/pF)	−7.3 ± 1.0	−1.0 ± 0.2	****	−0.4 ± 0.1	****	N/A
G_{max} (nS/nF)	131.4 ± 15.9	49.7 ± 6.6	***	22.6 ± 4.3	****	N/A
$V_{1/2}$ (mV)	2.4 ± 0.8	29.7 ± 2.9	****	24.0 ± 2.4	****	N/A
k_{act} (mV)	4.9 ± 0.3	13.1 ± 1.2	****	9.5 ± 0.9	0.002**	N/A
V_{rev} (mV)	73.7 ± 2.2	65.8 ± 4.8	0.24	59.3 ± 4.2	0.03*	N/A
Time to peak (ms)	61.4 ± 11.0	68.8 ± 7.4	0.86	98.2 ± 16.6	0.08	N/A
R_{500} (%)	64.6 ± 1.9	52.5 ± 11.3	0.45	73.3 ± 9.3	0.7	N/A
n	7	6	N/A	5	N/A	N/A
Parameters	$Ca_V1.2$ WT	$Ca_V1.2$ E318A	*p*-Value	N/A	N/A	N/A
I_{peak} (pA/pF)	−9.0 ± 0.9	−5.0 ± 0.7	***	N/A	N/A	N/A
G_{max} (nS/nF)	129.1 ± 16.0	70.3 ± 10.4	0.002**	N/A	N/A	N/A
$V_{1/2}$ (mV)	8.7 ± 2.3	14.1 ± 1.2	0.03*	N/A	N/A	N/A
k_{act} (mV)	6.3 ± 0.6	6.4 ± 0.4	0.86	N/A	N/A	N/A
V_{rev} (mV)	105.7 ± 7.0	105.4 ± 5.0	0.98	N/A	N/A	N/A
Time to peak (ms)	11.8 ± 1.8	13.0 ± 1.4	0.6	N/A	N/A	N/A
R_{500} (%)	59.9 ± 3.4	71.5 ± 3.3	0.02*	N/A	N/A	N/A
n	11	13	N/A	N/A	N/A	N/A

All data are presented as mean ± standard error. *p*-values were calculated using Student's *t*-test or ANOVA with Tukey's post hoc test. N/A, not applicable.
*$p < 0.05$
**$p < 0.01$
***$p \leq 0.001$
****$p \leq 0.0001$.

GFP-$Ca_V1.1e$-E216A (+15.7 mV) and GFP-$Ca_V1.1e$-E216Q (+6.8 mV) mirrors the different degrees of weakening the interactions with R1 and R2 by the alanine and glutamine substitutions shown in the structure models (Fig. 3, *A and B*).

E216 mutations do not further alter calcium channel gating properties in $Ca_V1.1a$

If indeed the effects on the gating properties of GFP-$Ca_V1.1e$ of the two substitutions of E216 in the PD of repeatI depend on the interdomain interaction of E216 with the gating charges in VSD IV, then this effect should be sensitive to structural perturbations in that VSD. Insertion of 19 amino acids encoded by exon 29 into the S3-S4 linker of VSD IV previously has been demonstrated to sever stabilizing ion-pair interactions of the gating charges within this VSD and to result in a 26–30 mV right shift of $V_{1/2}$ of activation. Consequently, in the presence of exon 29, mutation of D1196 had no further effect in $Ca_V1.1a$ (3,4,15). Are the effects of the E216 substitutions also abolished in $Ca_V1.1a$? In fact, homology modeling of $Ca_V1.1$ with and without exon 29 demonstrated that upon insertion of exon 29, the proposed interdomain interactions between E216 and R1 are already eliminated and those with R2 reduced to a single salt bridge (Fig. 2 *C*). Matching homology models of $Ca_V1.1a$-E216A and $Ca_V1.1a$-E216Q indicate that the two substitutions in IS5 have little further effects on the interactions with the gating charges in VSD IV

(Fig. 4, *A and B*). In $Ca_V1.1a$-E216A, the interaction with R2 is also lost. In $Ca_V1.1a$-E216Q, the remaining H-bond with R2 is maintained. The single ionic interaction between R2 and D1196 remaining after insertion of exon 29 is not affected by either substitution of E216.

To test the model predictions experimentally, we generated the corresponding mutant constructs in $Ca_V1.1a$ (GFP-$Ca_V1.1a$-E216A and GFP-$Ca_V1.1a$-E216Q), expressed them in dysgenic myotubes, and analyzed their current properties using patch-clamp recordings. As shown repeatedly before, the currents of wild-type $Ca_V1.1a$ channels were small and activated at substantially more positive potentials than $Ca_V1.1e$ (Table 1). This was also true for the E216A and E216Q mutants of $Ca_V1.1a$. Importantly, neither GFP-$Ca_V1.1a$-E216A (GFP-$Ca_V1.1a$: 33.6 ± 3.7 mV; GFP-$Ca_V1.1a$-E216A: 30.0 ± 3.3 mV; $p = 0.49$) (Fig. 4, *C–F*) nor GFP-$Ca_V1.1a$-E216Q (GFP-$Ca_V1.1e$: 34.7 ± 2.8 mV; GFP-$Ca_V1.1a$-E216A: 37.2 ± 2.7 mV; $p = 0.54$) (Fig. 4, *G–J*) showed a further right shift of the voltage dependence of activation compared to their wild-type controls (Fig. 4, *D–F and H–J*). This is consistent with the hypothesis that the right shift observed with the corresponding E216A/Q substitutions in $Ca_V1.1e$ is contingent on the regular function of VSD IV in the absence of exon 29.

The two putative ion-pair partners of the IVS4 gating charges cooperate in stabilizing the activated state of $Ca_V1.1e$

In $Ca_V1.1e$, charge neutralization of both E216 in IS5 or D1196 in IVS4 (4), individually, caused a right shift of the voltage dependence of activation. However, these effects were smaller than the right shifts produced by the insertion of exon 29 (Table 1) or by charge neutralization of R1 or R2 in IVS4 (4). This suggested that upon individual substitution, the preserved action of the respective other ion-pair partner partially sustained stabilization of VSD IV in the activated state. To examine whether the effects on $V_{1/2}$ are additive when both E216 and D1196 are neutralized simultaneously, we generated the double mutant GFP-$Ca_V1.1e$-E216A/D1196N and analyzed its gating properties. The structure models of VSD IV in the activated state demonstrate that these amino acid substitutions remove all ionic interactions with R1 and R2, although in the absence of E216

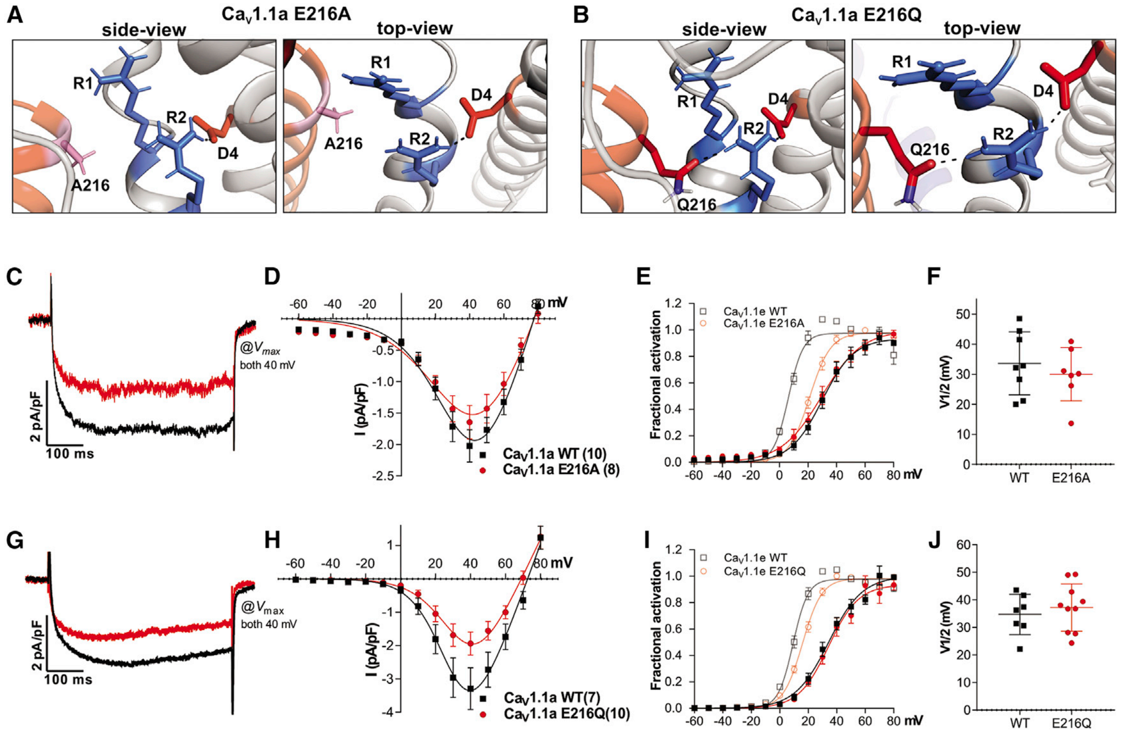

FIGURE 4 Mutations of E216 in $Ca_V1.1a$ (containing exon 29) have no further effect on channel gating properties. (*A* and *B*) Structures of the PD I – VSD IV interface of $Ca_V1.1a$ substitutions E216A (*A*) and E216Q (*B*), indicating the ion bonds and H-bonds (*dashed lines*) between the gating charges R1/R2 (*blue*), the countercharge D1196 (*red*), and the two substitutions for E216 (*pink/red*). Comparison with wild-type $Ca_V1.1a$ (c.f. Fig. 2 *C*) reveals no further reduction of interactions between the gating charges and the E216A/Q substitutions. (*C–F*) Representative example traces (*C*), I-V curves (*D*), and G-V curves (*E*) of calcium currents recorded from myotubes expressing wild-type GFP-$Ca_V1.1e$ (*black*) or GFP-$Ca_V1.1e$-E216A (*red*) compared to those of $Ca_V1.1e$ (*transparent*). (*F*) The E216A substitution causes no significant shift of $V_{1/2}$ (N = 7–8; $p = 0.49$). (*G–J*) Representative example traces (*G*), I-V curves (*H*), and G-V curves (*I*) of calcium currents recorded from myotubes expressing wild-type GFP-$Ca_V1.1a$ (*black*) or GFP-$Ca_V1.1a$-E216Q (*red*) compared to $Ca_V1.1e$ (*transparent*). (*J*) The E216Q substitution causes no significant shift of $V_{1/2}$ (N = 7–10; $p = 0.54$). P-values were calculated using a student t-test, error bars indicate standard error. For further current parameters, see Table 1. To see this figure in color, go online.

a weaker H-bond is formed between R2 and N1196 (Fig. 5 *A*). The current density in myotubes expressing GFP-$Ca_V1.1e$-E216A/D1196N was reduced to values below that of wild-type GFP-$Ca_V1.1a$ (Fig. 5, *B and C*). Considering the similar effects of GFP-$Ca_V1.1e$-D1196N (4), this was expected, but it also made analysis of current properties more challenging, as the currents in many cells were too small for reliable analysis. Nevertheless, the mean $V_{1/2}$ of the analyzable current recordings was right-shifted by 21.6 mV compared to the matched $Ca_V1.1e$ control, very close to the $V_{1/2}$ of wild-type $Ca_V1.1a$ recorded in parallel ($p = 0.2$) (Fig. 5 *D*). Thus, the current properties of GFP-$Ca_V1.1e$-E216A/D1196N roughly resemble those of $Ca_V1.1a$, and the effects of charge-neutralizing mutations of E216 and D1196 appear to be additive. This suggests that in $Ca_V1.1e$, the interdomain (E216) and intradomain (D1196) interactions cooperate in stabilizing VSD IV in the activated state and together constitute the molecular mechanism by which alternative splicing of exon 29 regulates the gating properties of $Ca_V1.1$ channels.

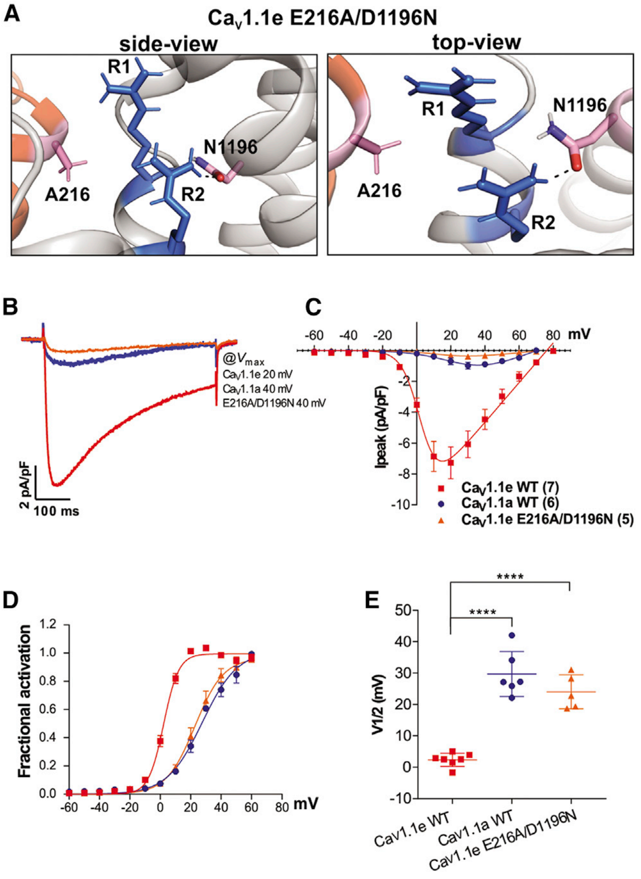

FIGURE 5 Double mutation of E216A and D1196N in $Ca_V1.1e$ reduces the current density and right shifts the activation curve toward that of $Ca_V1.1a$. (*A*) Side and top view of structures of the PD I – VSD IV interface of $Ca_V1.1e$ substitution E216A/D1196N, showing the total loss of ionic interactions, between the gating charges R1/R2 (*blue*) and the mutated countercharges N1196 and A216 (*pink*) (*dashed line*, remaining H-bond). (*B–E*) Representative example traces (*B*), I-V curves (*C*), and G-V curves (*D*) of calcium currents recorded from myotubes expressing wild-type GFP-$Ca_V1.1e$ (*red*), wild-type GFP-$Ca_V1.1a$ (*blue*), or GFP-$Ca_V1.1e$-E216A/D1196N (*orange*). (*E*) The simultaneous E216A and D1196N substitution causes a significant right shift of $V_{1/2}$ of 21.6 mV (N = 5–7; $p < 0.0001$). No significant difference in $V_{1/2}$ was observed between wild-type $Ca_V1.1a$ and $Ca_V1.1e$-E216A/D1196N ($p = 0.2$). P-values were calculated using an ANOVA with Tukey's posthoc test, error bars indicate standard error. For further current parameters, see Table 1. To see this figure in color, go online.

Interdomain interactions between outer gating charges of IVS4 with an ion-pair partner in IS5 are specific for $Ca_V1.1$ and $Ca_V1.2$ channels

Given the evidence in support of an important contribution of this interdomain interaction in regulating $Ca_V1.1$ gating properties, we wondered whether this is a specific feature of the skeletal muscle channel isoform, which is uniquely sensitive to alternative splicing in the IVS3-S4 linker (28), or whether this regulatory molecular mechanism is more broadly realized in the Ca_V channel family. Among the 10 Ca_V family members, the negatively charged glutamate in the corresponding position in IS5 is conserved in all Ca_V1 and Ca_V2 channels (Fig. S2 *A*). In contrast, Ca_V3 channels contain an uncharged glutamine in the corresponding position of IS5. Thus, an ionic interaction between the PD of the first repeat and outer gating charges in VSD IV, similar to that observed in $Ca_V1.1$, would be possible in all high-voltage-activated channels, but not in low-voltage-activated channels. Nevertheless, homology modeling indicated the formation of ion pairs with the outer gating charges of VSD IV only in two members of the Ca_V1 family: $Ca_V1.1$ and $Ca_V1.2$ (Fig. S2, *C–G*). Between these two channels, the MD simulation suggested a substantially reduced probability of this interdomain interaction in $Ca_V1.2$ compared to $Ca_V1.1e$, which is reflected in a substantially smaller incidence of this ionic interaction in $Ca_V1.2$ (34%) compared to $Ca_V1.1e$ (73%). To further estimate the stability of the interaction, we calculated the linear interaction energy. Indeed, the reduced probability of this interaction in $Ca_V1.2$ relative to $Ca_V1.1e$ is accompanied by a weaker interaction of R1 and R2 with E318. For $Ca_V1.2$, the electrostatic linear interaction energy was −80.2 kcal/mol, compared to −120.4 kcal/mol in $Ca_V1.1e$. $Ca_V1.1a$ and the channels with missing or disrupted ion pairs had substantially lower energies (Table S1).

Because the structure models suggested the existence of this interdomain ion-pair interaction in $Ca_V1.2$ (Fig. 6, *A and B*), we experimentally tested whether the putative countercharge in IS5 is of functional importance for $Ca_V1.2$ channel gating. Therefore, we substituted the corresponding amino acid (E318) in $Ca_V1.2$ with an alanine (GFP-$Ca_V1.2$-E318A) and analyzed the construct in dysgenic myotubes. Immunofluorescence labeling confirmed that wild-type and mutant $Ca_V1.2$ channels were equally expressed and targeted into the triad junctions (Fig. S5). Fig. 6, *C–F* show that the patch-clamp analysis of GFP-$Ca_V1.2$-E318A resulted in

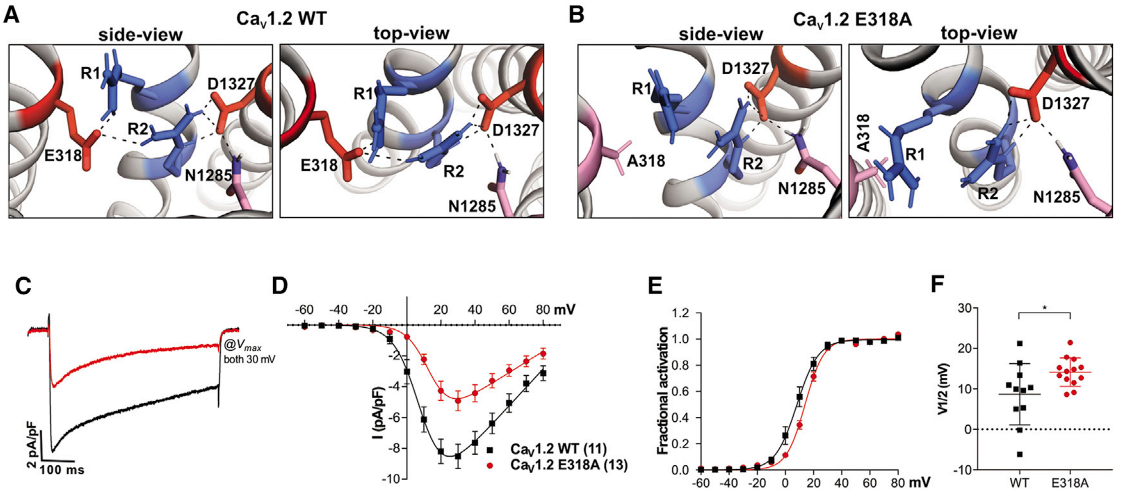

FIGURE 6 $Ca_V1.2$ E318 (corresponding to E216 in $Ca_V1.1$) interacts with outer gating charges of VSD IV and participates in the regulation of gating properties. (*A* and *B*) Structures of the PD I – VSD IV interface of $Ca_V1.2$ WT (*A*) and the $Ca_V1.2$-E318A mutant (*B*) indicating the ion bonds and H-bonds (*dashed lines*) between the gating charges R1/R2 (*blue*) and the countercharges D1327 (*red*) in IVS3 and E318 (*red/pink*) in IS5. Charge neutralization in E318A abolishes all stabilizing interactions with R1, whereas R2 remains stabilized by its multiple interactions with D1327. (*C–E*) Representative example traces (*C*), I-V curves (*D*), and G-V curves (*E*) of calcium currents recorded from myotubes expressing wild-type GFP-$Ca_V1.2$ (*black*) or GFP-$Ca_V1.2$-E318A (*red*). (*F*) The E318A substitution causes a significant right shift of $V_{1/2}$ ($N = 11–13$; $p = 0.03$). For further current parameters, see Table 1. To see this figure in color, go online.

currents of reduced amplitude, slightly faster inactivation, and importantly a small but significant right shift in the voltage dependence of activation compared to wild-type controls (GFP-$Ca_V1.2$: 8.7 $\pm$ 2.3 mV; GFP-$Ca_V1.2$-E318A: 14.1 $\pm$ 1.2 mV; $p = 0.03$). The smaller magnitude of the effect matches the reduced propensity to form such ion-pair interactions in $Ca_V1.2$ compared to $Ca_V1.1$e observed in the structure model. Together, modeling and analysis of gating properties suggest the possibility of such an interdomain interaction also in $Ca_V1.2$, but with a lesser functional relevance compared to that in $Ca_V1.1$.

DISCUSSION

The results presented here demonstrate that in $Ca_V1.1$e, the conserved negatively charged glutamate E216 in the PD of the first repeat forms interdomain ion pairs with the outer gating charges R1 and R2 of the VSD in the fourth repeat to stabilize this VSD in the activated conformation and thus facilitate channel gating. Multiple lines of evidence support this conclusion: first, structure modeling identified interactions between E216 and R1 and R2 of VSD IV in the activated state of $Ca_V1.1$e. Secondly, amino acid substitutions that remove the positive charge (E216Q) or curtail the entire side chain (E216A) weakened or removed, respectively, the interactions with the gating charges and caused a shift of the voltage dependence of activation to more positive potentials. Thirdly, the size of this right shift of $V_{1/2}$ was greater for E216A than for E216Q, thus corresponding to the number of interactions removed by the two substitutions observed in the structure models. Finally, insertion of exon 29 into the S3-S4 linker of VSD IV severed the interdomain interactions between E216 and R1/R2 and abolished the effects of the amino acid substitutions in PD I on channel gating.

This behavior of E216 in these experiments is reminiscent of that previously observed for D1196, an ion-pair partner of R1 and R2 within VSD IV. These intra-VSD interactions and their importance for determining the voltage dependence of activation were first observed in a homology structure model of the isolated VSD IV (4). In the present structure model, based on the experimentally determined structure of the whole $Ca_V1.1$ (11), we confirm these interactions. D1196 forms two H-bonds each with R1 and R2. Consequently, R1 and R2 form twice as many intra-VSD interactions with D1196 as interdomain interactions with E216. In line with these different contributions of the two ion-pair partners, the magnitude of the right shift of $V_{1/2}$ obtained with substitutions of D1196 was substantially larger (~20 mV for D1196N or D1196E) than that reported here for E216A (15.7 mV) and E216Q (6.8 mV) (Fig. 7). Together, the correlation between the number and the strength of the interactions of R1 and R2 in IVS4 with its two ion-pair partners and the magnitude of the effects on $V_{1/2}$ validate our structure model and strongly support the proposed function of these interactions in stabilizing the activated state of $Ca_V1.1$e. This notion is further corroborated by our finding that insertion of the 19 amino acids of exon 29 into the IVS3-S4 linker abolishes most of these interactions and by itself right shifts $V_{1/2}$.

In addition to the right-shifted $V_{1/2}$, substitutions of D1196 or disruption of its interactions with R1 and R2 by insertion of exon 29 also caused a severalfold decrease in current density (4,15). However, a similar effect on current density was not the seen here when E216 was mutated. This difference might in part be explained by the higher number

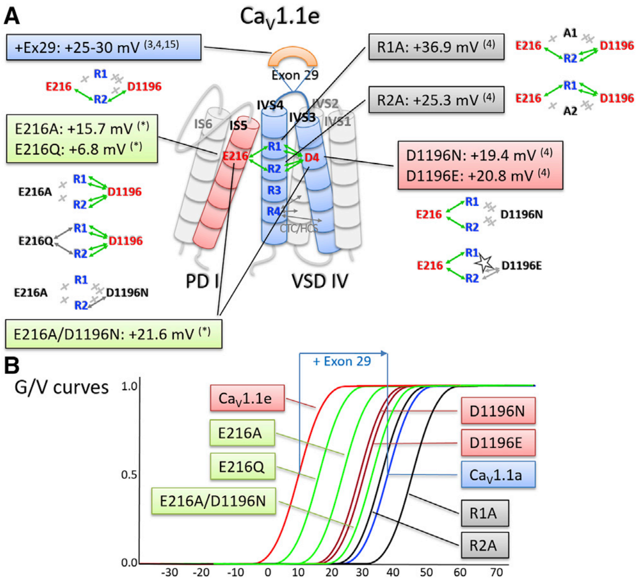

FIGURE 7 Gating charge interactions of R1 and R2 in VSD IV with ion-pair partners E216 in IS5 and D1196 in IVS3 and the effects of their mutations on the voltage dependence of activation. (*A*) Cylinder model of VSD IV and adjacent PD I displaying the ionic interactions (*green double arrows*) of R1/R2 with E216 and D1169 (D4). Colored boxes in (*A*) and schematic G-V curves in (*B*) show the magnitude of the right shift of the voltage dependence of activation by mutation of the individual interaction partners and by insertion of exon 29. Values are from this (*) and previously published studies (3,4,15). Cartoons below the boxes in (*A*) indicate the effects of the mutations on the number and strength of the interactions observed in the structure models of the mutated channels (see Figs. 2 *C*; 3, *A* and *B*; 4, *A* and *B*; 5 *A*; and S2): Green arrows, ionic bonds; gray arrows, H-bonds; gray x and stars, abolished interactions and structural clashes, respectively. To see this figure in color, go online.

of interactions formed between R1/R2 and D1196, compared to E216.

Structurally, formation of interdomain interactions between residues at the outer end of S4 and E216 involved in stabilizing the activated state is highly plausible. In the bacterial Na_VAb channel, thought to be ancestral to Ca_Vs and Na_Vs, screening of amino acid positions capable of forming disulfide links between S4 and S5 of adjacent channel monomers identified Q150 in the S5 helix (29). This glutamine is highly conserved in the VSD of eukaryotic Ca_V channels, except in VSD I of Ca_V1 and Ca_V2 channels, in which it is replaced by a glutamate, E216 in $Ca_V1.1$. In line with our findings, replacing Q150 with cysteine and chemical cross-linking with a second cysteine inserted in the position of R1 in the S4 helix stabilized the Na_VAb tetramers and the activated state of the channel. In eukaryotic high-voltage-gated calcium channels, substitution of the glutamine in this favorable position with a negatively charged glutamate appears to do the same thing naturally. E216 enables the formation of interdomain ion pairs with the outer gating charges and thus contributes to stabilizing the channel in the activated state.

Interdomain interactions between VSDs and PDs, specifically between amino acid residues in the S4 and S5 helices, have also been observed in the *Shaker* potassium channel (30,31). These function in a non-canonical coupling mechanism between VSDs and the PDs of adjacent subunits and, together with the canonical coupling of VSDs and PDs of the same subunit via the S4-S5 linker, provide voltage sensitivity to gating of the channel pore. It has been suggested that such a non-canonical coupling might be a general principle in domain-swapped channels, including Ca_Vs (30). In theory, the interaction between R1 and R2 and E216 described here might be part of such a non-canonical coupling mechanism, and this possibility deserves to be examined in future studies. However, the R1/R2-E216 interaction reported here differs in several aspects from the non-canonical coupling mechanism of *Shaker* channels. We report ionic interactions of the two outer gating charges in IVS4 with a negative countercharge in IS5 specifically in the activated state. In contrast, noncanonical coupling in *Shaker* utilizes the sequential interactions of three hydrophobic residues interspersed with the S4 gating charges and a polar serine in S5 in resting and the activated states. In the homotetrameric potassium channel, this coupling at the VSD-PD interface occurs in every subunit, whereas in $Ca_V1.1$, the interaction with E216 is unique for the interface between VSD IV and PD I. Finally, a general mechanism in domain-swapped channels would be expected to be conserved throughout a channel family, whereas the R1/R2-E216 interaction described here is structurally and functionally specific for $Ca_V1.1$ and $Ca_V1.2$ channels. Therefore, although we cannot exclude the possibility that in $Ca_V1.1$ this interdomain interaction is involved in transmitting the action of VSD IV to gating of the channel pore, its primary importance most likely is in stabilizing IVS4 in the activated state and in the modulation of this process by alternative splicing of exon 29.

Together with the results from our previous studies of $Ca_V1.1$ VSD IV function, our data suggest the following mechanism (Fig. 7): in wild-type $Ca_V1.1e$, the extracellular end of the IVS4 helix is stabilized in the activated state by simultaneous interactions of the R1 and R2 gating charges with two ion-pair partners, D1196 in IVS3 and E216 in IS5. Both of them are essential for efficient voltage-sensor function and channel gating in the embryonic splice variant ($Ca_V1.1e$). Consistent with this model, mutation of one or the other ion-pair partner shifts the voltage dependence of activation to more positive potentials. This effect is larger for substitutions of D1196 (~20 mV) than for substitution of E216 (~16 mV). Importantly, the different magnitude of the functional effects corresponds well to the number of interactions formed between R1/R2 and the two ion-pair partners observed in the structures. D1196 forms two interactions each with R1 and R2, and E216 forms one interaction each with R1 and R2. Moreover, when the interactions with E216 are reduced to a single H-bond (in E216Q) the magnitude of the right shift is halved. This suggests that the number and strength of the interactions with

the two countercharges determine the degree of stabilization of the activated state. Furthermore, the effects of substituting either one of the two ion-pair partners (E216A/Q, D1196N/E) are smaller than the effects of mutating the gating charges R1 or R2 (R1A, 36 mV; R2A, 25 mV (4)). This indicates that the effects of both ion-pair partners in stabilizing the activated state are additive. Accordingly, structure modeling of the mutants showed that the substitution of either one of the two countercharges does not perturb the respective other. In E216A, the interactions between D1196 and R1/R2 were maintained. Similarly, in D1196N the interactions between E216 and R1/R2 were maintained (Fig. S3). Only the combined mutation of E216 and D1196 abolished all ionic interactions with R1 and R2 and right-shifted $V_{1/2}$ further than the individual substitutions and also reduced the current density to values similar to those of $Ca_V1.1a$. Overall, the voltage dependence of activation of the embryonic $Ca_V1.1e$ splice variant is critically regulated by the sum of ionic interactions formed, in the activated state, between the outer gating charges R1/R2 and the two ion-pair partners within (D1196) and outside of VSD IV (E216).

Because the two countercharges are positioned at the extracellular ends of IVS3 and IS5, their position relative to IVS4 and their interactions formed with R1/R2 are highly susceptible to structural alterations in the extracellular loop connecting IVS3 and IVS4. In the adult $Ca_V1.1a$ splice variant, which contains exon 29 in the IVS3-S4 loop, four of the six interactions are lost. Only one interaction each of R2 with D1196 and E216 remains intact. Functionally, insertion of exon 29 results in a substantial right shift of the voltage dependence of activation (25–30 mV (3,4,15)). Consistent with the notion that the right shift of $V_{1/2}$ in response to mutating E216 (or D1196) in $Ca_V1.1e$ and in response to insertion of exon 29 in $Ca_V1.1a$ resulted from eliminating the same interactions with R1/R2, the corresponding mutations of E216 (or D1196) had no further effect on $V_{1/2}$ in $Ca_V1.1a$. Alternatively, the absence of measurable effects in $Ca_V1.1a$ might be explained if the relatively smaller shifts of $V_{1/2}$ in the mutations were masked by the large shift of $V_{1/2}$ caused by alternative splicing. However, previously we demonstrated that this is not necessarily the case. Even a modest right shift of $V_{1/2}$ resulting from a mutation unrelated to the function of VSD IV could be observed in $Ca_V1.1e$ as well as in $Ca_V1.1a$ (32). Thus, further right shifting $V_{1/2}$ in $Ca_V1.1a$ is possible, and there is no reason to assume that the splicing-dependent shift of $V_{1/2}$ would mask the effects of the substitutions of E216 (or D1196). Consequently, eliminating possible interactions with R1/R2 by mutating E216 (or D1196) did not affect the voltage dependence of activation in $Ca_V1.1a$ because the critical stabilizing interactions had already been removed by the insertion of exon 29.

Consistent with the particular importance of VSD IV in regulating the characteristic voltage dependences of activation of $Ca_V1.1$, the reported interdomain interaction is unique for the interface between PD I and VSD IV. The other VSD-PD interfaces (IIS5, IIIS5, and IVS5) contain uncharged glutamines in the position corresponding to E216 in IS5, and the structure models did not reveal any interactions with gating charges. Recently, we demonstrated that apart from the described interactions with countercharges in the activated state, the gating charges and the ENC of VSD IV form few, if any, ion pairs in the resting states (7). Also, the interactions with E216 are reduced to a single salt bridge with R1 in resting state 3 (Fig. S6) and completely lost as IVS4 moves further downward to resting states 2 and 1. This is in stark contrast to the other VSDs. For example, in VSD I, the gating charges form multiple stabilizing interactions sequentially in the resting states and thereby endow $Ca_V1.1$ with its characteristic slow activation kinetics (7). The unique existence of the interdomain interaction with E216 in VSD IV further emphasizes the structural and functional differences between the four VSDs in Ca_V channels.

In addition to E216 in $Ca_V1.1$, a structurally and functionally homologous interdomain interaction was also found in $Ca_V1.2$. There, the right shift in $V_{1/2}$ produced by the corresponding E318A mutation was smaller than that in $Ca_V1.1$, matching the reduced probability at which such ion pairs were observed in the MD simulation. Interestingly, the relative smaller right shift of $V_{1/2}$ in $Ca_V1.2$ corresponds well to the relatively smaller right shift of $V_{1/2}$ observed upon alternative splicing of its exon 33 (27,28), suggesting that this mechanism to regulate the voltage dependence of the two Ca_V1 channels is related to their facility to be regulated by alternative splicing in the IVS3-S4 linker. The absence of similar activation-stabilizing interdomain interactions in other VSDs and in other Ca_V channels shows that not only are the four VSD of Ca_V channels highly distinct from each other, but they also differ substantially from the corresponding VSDs in other members of this channel family. Conversely, our results highlight how the individual VSDs of Ca_V channels are uniquely tailored to generate the specific gating properties of each channel isoform and splice variant and that ion-pair interactions between the outer gating charges and countercharges in the ENC play an important role in this process.

CONCLUSIONS

To our knowledge, this is the first report of a functional molecular interaction between a VSD and the adjacent PD in a voltage-gated calcium channel. Specifically, in $Ca_V1.1$ (and also in $Ca_V1.2$), the two outer gating charges R1 and R2 of IVS4 form ionic interactions with a countercharge E216 located in IS5. This interdomain interaction cooperates with additional ionic interactions within VSD IV in stabilizing the activated state of the channel. The number and strength of these ion-pair interactions are the critical determinants of

$Ca_V1.1$'s voltage dependence of activation and create a mechanism susceptible to regulation by alternative splicing.

SUPPORTING MATERIAL

Supporting material can be found online at https://doi.org/10.1016/j.bpj.2021.09.004.

AUTHOR CONTRIBUTIONS

B.E.F. conceived and supervised the project. Y.E.G., M.L.F.-Q., S.M., and M.C. planned and performed the computer simulations and/or experiments and analyzed the data. P.T. and K.R.L. planned and supervised specific aspects of the experiments and data analyses. B.E.F. wrote the manuscript, and all authors participated in discussion of results, manuscript preparation, and editing.

ACKNOWLEDGMENTS

We thank I. Mahlknecht, M. Heitz, and S. Demetz for excellent technical support. The computational data have been obtained in part using the Vienna Scientific Cluster.

The work was supported by funds from the Austrian Science Fund P30402 and DOC30 (to B.E.F.).

REFERENCES

1. Catterall, W. A., M. J. Lenaeus, and T. M. Gamal El-Din. 2020. Structure and pharmacology of voltage-gated sodium and calcium channels. *Annu. Rev. Pharmacol. Toxicol.* 60:133–154.
2. Chanda, B., and F. Bezanilla. 2008. A common pathway for charge transport through voltage-sensing domains. *Neuron.* 57:345–351.
3. Tuluc, P., B. Benedetti, …, B. E. Flucher. 2016. Two distinct voltage-sensing domains control voltage sensitivity and kinetics of current activation in CaV1.1 calcium channels. *J. Gen. Physiol.* 147:437–449.
4. Tuluc, P., V. Yarov-Yarovoy, …, B. E. Flucher. 2016. Molecular interactions in the voltage sensor controlling gating properties of CaV calcium channels. *Structure.* 24:261–271.
5. Catterall, W. A., G. Wisedchaisri, and N. Zheng. 2017. The chemical basis for electrical signaling. *Nat. Chem. Biol.* 13:455–463.
6. Groome, J. R., and L. Bayless-Edwards. 2020. Roles for countercharge in the voltage sensor domain of ion channels. *Front. Pharmacol.* 11:160.
7. Fernández-Quintero, M. L., Y. El Ghaleb, …, B. E. Flucher. 2021. Structural determinants of voltage-gating properties in calcium channels. *eLife.* 10:e64087.
8. Payandeh, J., T. Scheuer, …, W. A. Catterall. 2011. The crystal structure of a voltage-gated sodium channel. *Nature.* 475:353–358.
9. Ahern, C. A., J. Payandeh, …, B. Chanda. 2016. The hitchhiker's guide to the voltage-gated sodium channel galaxy. *J. Gen. Physiol.* 147:1–24.
10. Pantazis, A., N. Savalli, …, R. Olcese. 2014. Functional heterogeneity of the four voltage sensors of a human L-type calcium channel. *Proc. Natl. Acad. Sci. USA.* 111:18381–18386.
11. Wu, J., Z. Yan, …, N. Yan. 2016. Structure of the voltage-gated calcium channel Ca(v)1.1 at 3.6 Å resolution. *Nature.* 537:191–196.
12. Flucher, B. E., and P. Tuluc. 2017. How and why are calcium currents curtailed in the skeletal muscle voltage-gated calcium channels? *J. Physiol.* 595:1451–1463.
13. Melzer, W., A. Herrmann-Frank, and H. C. Lüttgau. 1995. The role of Ca2+ ions in excitation-contraction coupling of skeletal muscle fibres. *Biochim. Biophys. Acta.* 1241:59–116.
14. Nakai, J., B. A. Adams, …, K. G. Beam. 1994. Critical roles of the S3 segment and S3-S4 linker of repeat I in activation of L-type calcium channels. *Proc. Natl. Acad. Sci. USA.* 91:1014–1018.
15. Tuluc, P., N. Molenda, …, K. Jurkat-Rott. 2009. A CaV1.1 Ca2+ channel splice variant with high conductance and voltage-sensitivity alters EC coupling in developing skeletal muscle. *Biophys. J.* 96:35–44.
16. Rohl, C. A., C. E. M. Strauss, …, D. Baker. 2004. Protein structure prediction using Rosetta. *Methods Enzymol.* 383:66–93.
17. Zhao, Y., G. Huang, …, N. Yan. 2019. Cryo-EM structures of apo and antagonist-bound human $Ca_v3.1$. *Nature.* 576:492–497.
18. Lee, J., D. S. Patel, …, W. Im. 2019. CHARMM-GUI *Membrane Builder* for complex biological membrane simulations with glycolipids and lipoglycans. *J. Chem. Theory Comput.* 15:775–786.
19. Jo, S., J. B. Lim, …, W. Im. 2009. CHARMM-GUI Membrane Builder for mixed bilayers and its application to yeast membranes. *Biophys. J.* 97:50–58.
20. Case, D. A., I. Y. Ben-Shalom, …, P. A. Kollman. 2018. AMBER 2018. University of California, San Francisco, CA.
21. Dickson, C. J., B. D. Madej, …, R. C. Walker. 2014. Lipid14: the Amber lipid force field. *J. Chem. Theory Comput.* 10:865–879.
22. Salomon-Ferrer, R., A. W. Götz, …, R. C. Walker. 2013. Routine microsecond molecular dynamics simulations with AMBER on GPUs. 2. Explicit solvent Particle Mesh Ewald. *J. Chem. Theory Comput.* 9:3878–3888.
23. Grabner, M., R. T. Dirksen, and K. G. Beam. 1998. Tagging with green fluorescent protein reveals a distinct subcellular distribution of L-type and non-L-type Ca2+ channels expressed in dysgenic myotubes. *Proc. Natl. Acad. Sci. USA.* 95:1903–1908.
24. Powell, J. A., L. Petherbridge, and B. E. Flucher. 1996. Formation of triads without the dihydropyridine receptor alpha subunits in cell lines from dysgenic skeletal muscle. *J. Cell Biol.* 134:375–387.
25. Flucher, B. E., S. B. Andrews, and M. P. Daniels. 1994. Molecular organization of transverse tubule/sarcoplasmic reticulum junctions during development of excitation-contraction coupling in skeletal muscle. *Mol. Biol. Cell.* 5:1105–1118.
26. Fuchs, J. E., B. J. Waldner, …, K. R. Liedl. 2015. Independent metrics for protein backbone and side-chain flexibility: time scales and effects of ligand binding. *J. Chem. Theory Comput.* 11:851–860.
27. Costé de Bagneaux, P., M. Campiglio, …, B. E. Flucher. 2018. Role of putative voltage-sensor countercharge D4 in regulating gating properties of $Ca_V1.2$ and $Ca_V1.3$ calcium channels. *Channels (Austin).* 12:249–261.
28. Liu, N., Y. Liu, …, X. Liu. 2016. Linker flexibility of IVS3-S4 loops modulates voltage-dependent activation of L-type Ca 2+ channels. *Channels (Austin).* 11:34–45.
29. Wisedchaisri, G., L. Tonggu, …, W. A. Catterall. 2019. Resting-state structure and gating mechanism of a voltage-gated sodium channel. *Cell.* 178:993–1003.e12.
30. Carvalho-de-Souza, J. L., and F. Bezanilla. 2019. Noncanonical mechanism of voltage sensor coupling to pore revealed by tandem dimers of Shaker. *Nat. Commun.* 10:3584.
31. Fernández-Mariño, A. I., T. J. Harpole, …, B. Chanda. 2018. Gating interaction maps reveal a noncanonical electromechanical coupling mode in the Shaker K^+ channel. *Nat. Struct. Mol. Biol.* 25:320–326.
32. El Ghaleb, Y., M. Campiglio, and B. E. Flucher. 2019. Correcting the R165K substitution in the first voltage-sensor of $Ca_V1.1$ right-shifts the voltage-dependence of skeletal muscle calcium channel activation. *Channels (Austin).* 13:62–71.

Biophysical Journal, Volume *120*

Supplemental information

Ion-pair interactions between voltage-sensing domain IV and pore domain I regulate Ca_V1.1 gating

Yousra El Ghaleb, Monica L. Fernández-Quintero, Stefania Monteleone, Petronel Tuluc, Marta Campiglio, Klaus R. Liedl, and Bernhard E. Flucher

A

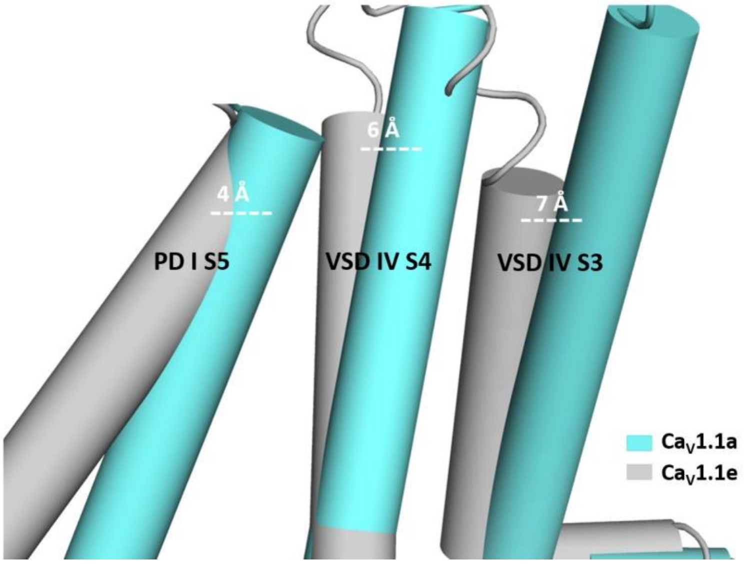

B

Cα-distance (Å)	Ca$_V$1.1a	Ca$_V$1.1e	Δ
**E216 - R1**	6.6	7.8	1.2
**D4 - R1**	6.4	5.4	1.0

Figure S1. Insertion of exon 29 in the S3-S4 linker of VSD IV causes a displacement of the α-helices and changes in the relative position of the Cα-atoms of the ion pair partners. (**A**) Cylinder-model showing IS5, IVS3 and IVS4 of Ca$_V$1.1e (lacking exon 29, gray) and Ca$_V$1.1a (including exon 29, turquoise) derived from overlays of the structure models. Displacement distances of the Cα-atoms of E216, R1, and D1196 are shown for the two splice variants. (**B**) Table showing the displacement and the resulting changes in the distance between the ion pair partners.

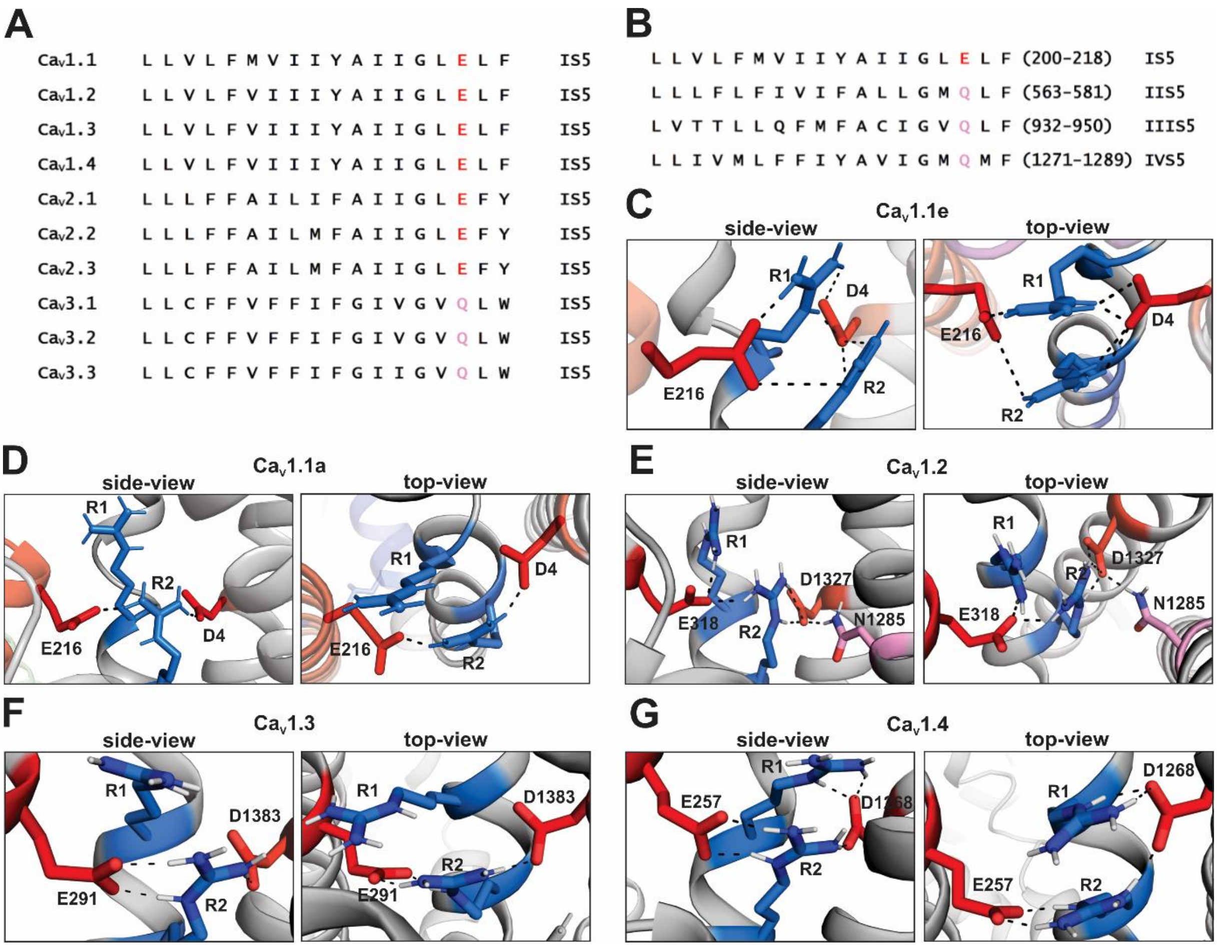

Figure S2. The inter-domain interaction between VSD IV and IS5 is specific for Ca$_V$1.1 and Ca$_V$1.2, and in Ca$_V$1.1 specific for IS5. (**A**) Sequence alignment of the IS5 helix of all ten Ca$_V$ family members (human sequences). Across the high-voltage-activated channels (Ca$_V$1 and Ca$_V$2) the sequence identity in IS5 is very high and the negatively charged glutamate (red) corresponding to E216 in Ca$_V$1.1 is fully conserved. In contrast, the low-voltage-gated Ca$_V$3 channels contain an uncharged glutamine (pink) in the corresponding position. (**B**) Sequence alignment of the four S5 segments in Ca$_V$1.1 shows that the glutamate residue E216 (red) is specific for the first repeat (IS5), while the other three repeats (IIS5, IIIS5, IVS5) contain a glutamine (pink) in the corresponding position. Thus, conservation across the Ca$_V$ genes is much higher than in the four repeats of Ca$_V$1.1, and the inter-domain ion-pair interaction reported here can only occur between VSD IV and PD I. (**C**) In Ca$_V$1.1e E216 forms ion pairs with both R1 and R2. (**D**) In contrast, in Ca$_V$1.1a the ion-pair of E216 with R1 is broken. (**E**) Comparable to the situation in Ca$_V$1.1e, E318 in Ca$_V$1.2 interacts with both R1 and R2. (**F, G**) In Ca$_V$1.3 E291 and in Ca$_V$1.4 E257 interact only with R2 and not with R1, similar to the situation in Ca$_V$1.1a.

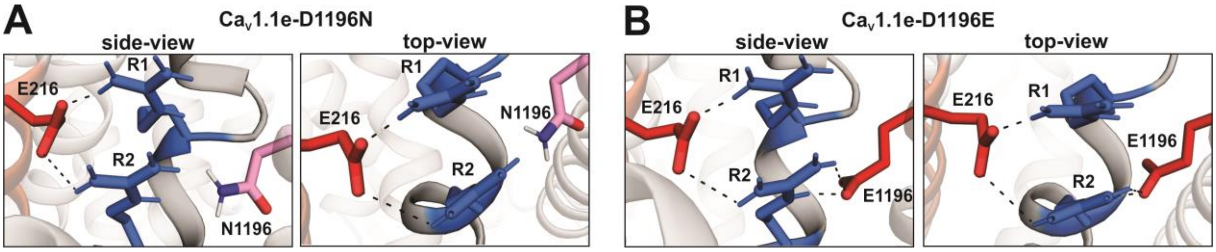

Figure S3. Substitutions of the intra-domain ion-pair partner D1196 eliminate stabilizing interactions with R1 and R2 in Ca_V1.1e. Previously we identified D1196 in IVS3 as a critical intra-domain ion-pair partner stabilizing R1 and R2 of IVS4 in the activated state (15). Substitution of D1196 with N or E caused a substantial right-shift of V½. Homology models of the complete Ca_V1.1e containing these amino acid substitutions shows that in the charge-neutralizing mutation Ca_V1.1e-D1196N the ionic interactions with R1 and R2 are abolished (**A**) and that in the charge-maintaining mutation Ca_V1.1e-D1196E the ionic interactions with R1 is severed as the result of clashes produced by the increased sidechain length (**B**). Importantly, severing these intra-domain interactions does not perturb the inter-domain interactions of R1 and R1 with E216.

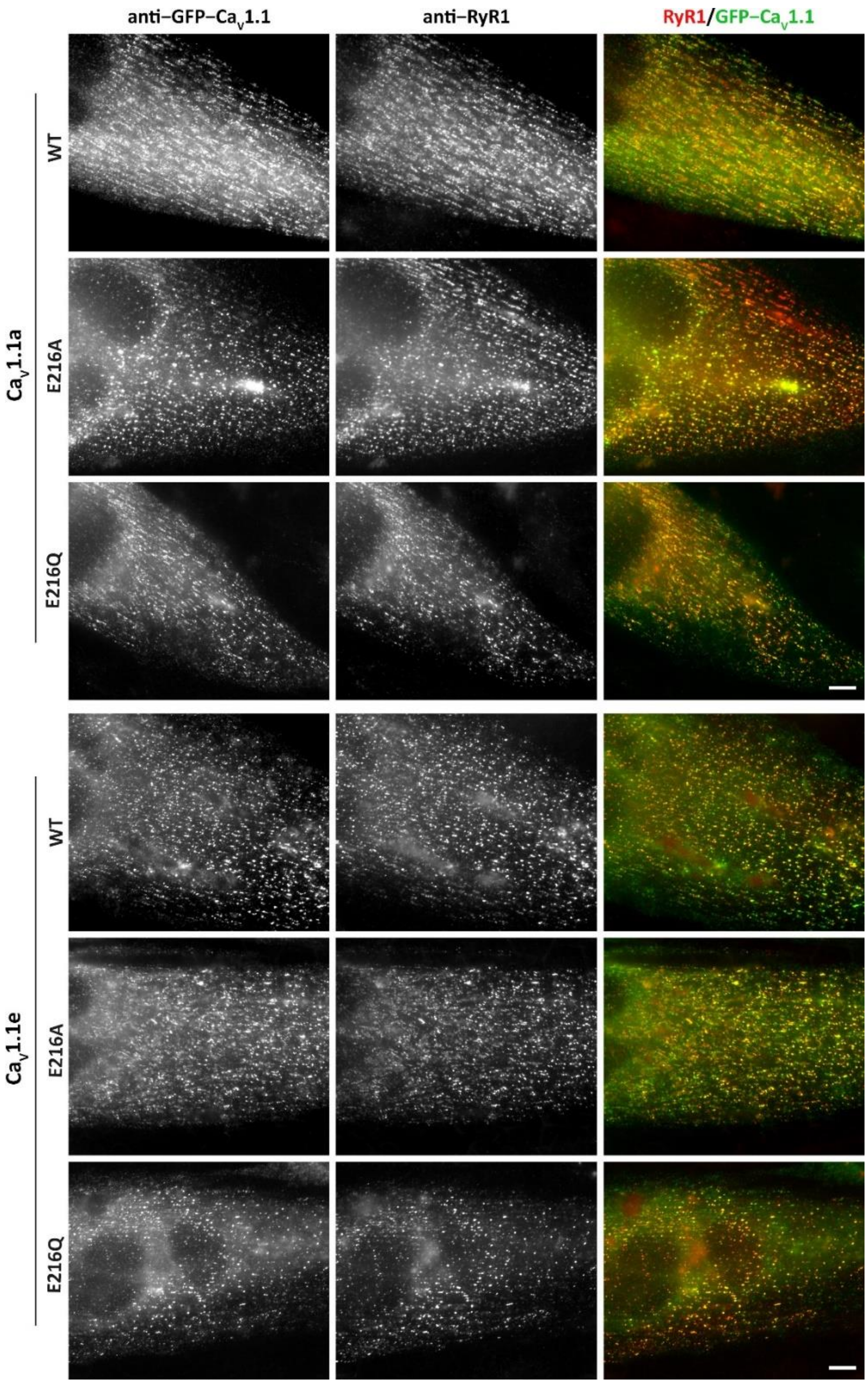

Figure S4: Expression and triad targeting of WT GFP-Ca$_V$1.1a and GFP-Ca$_V$1.1e channels and the E216A and E216Q mutant channels in dysgenic myotubes. Dysgenic (Ca$_V$1.1-null) myotubes were transfected with the indicated GFP-Ca$_V$1.1 constructs and myotubes were fixed and double-immunofluorescence labeled with anti-GFP (to localize the Ca$_V$1.1 construct) and anti-RyR1 (as a triad marker). Colocalization of GFP-Ca$_V$1.1 constructs with RyR1 in clusters indicates its normal incorporation into triadic calcium channel complexes of skeletal myotubes. The E216A and E216Q mutants showed similar expression and distribution as the wildtype GFP-Ca$_V$1.1a and Ca$_V$1.1e. Scale bars, 10 µm.

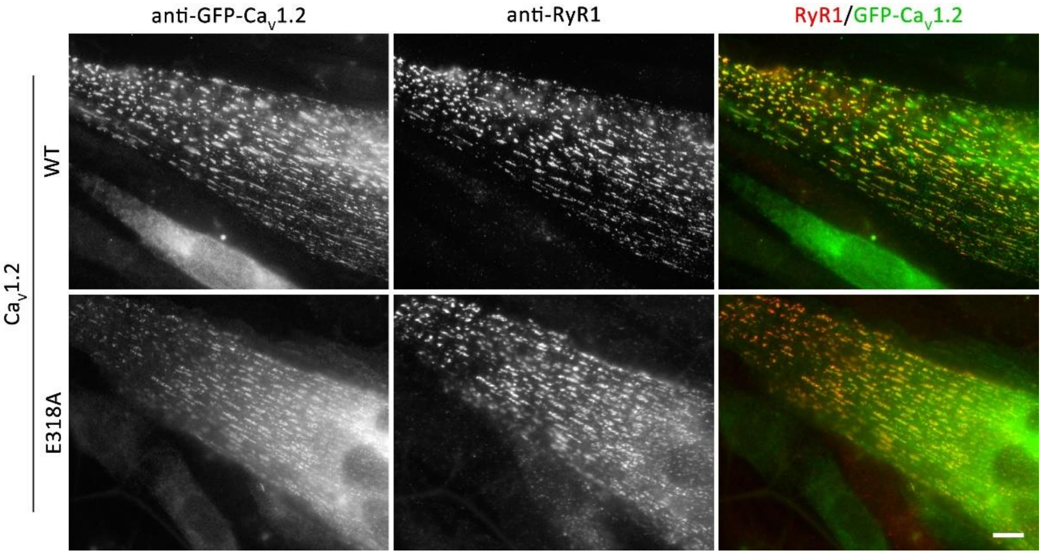

Figure S5. Expression and triad targeting of WT and E318A mutant GFP-Ca_V1.2 channels in dysgenic myotubes. Dysgenic (Ca_V1.1-null) myotubes were transfected with the indicated GFP-Ca_V1.2 constructs and myotubes were fixed and double-immunofluorescence labeled with anti-GFP (to localize the Ca_V1.2 construct) and anti-RyR1 (as a triad marker). Colocalization of GFP-Ca_V1.2 constructs with RyR1 in clusters indicates its normal incorporation into triadic calcium channel complexes of skeletal myotubes. GFP-Ca_V1.2-E318A showed similar expression and distribution as the wildtype GFP-Ca_V1.2. Scale bars, 10 µm.

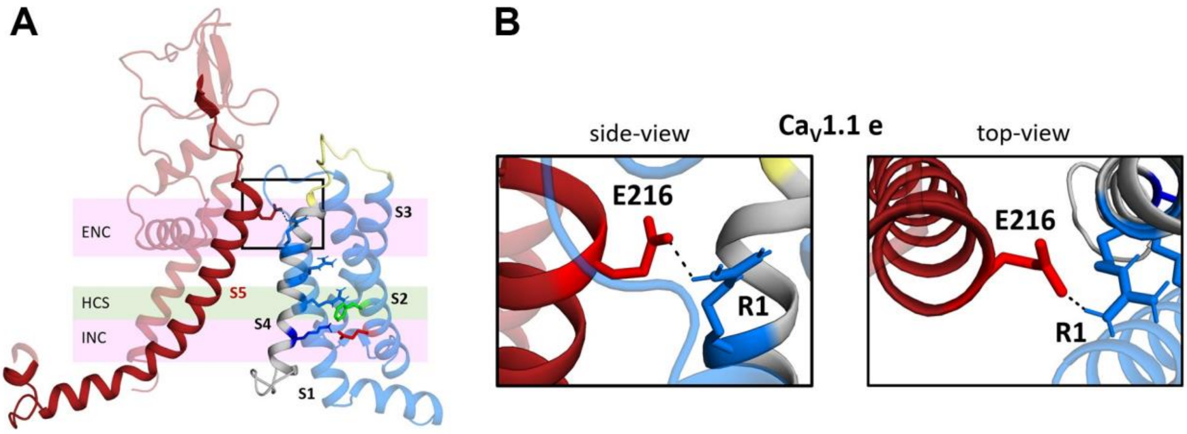

Figure S6. Reduced inter-domain interactions between IVS4 and IS5 in resting state 3 of Ca_V1.1e. The recently published resting state 3 structure model of VSD IV (5) was used to characterize the influence of the sliding S4 helix on the inter-domain interaction with IS5. In resting state 3 the S4 helix has moved down one step from the activated state. The resting state 3 model of VSD IV in Ca_V1.1e was obtained by aligning the trans-membrane helices S1-S3 with the VSD IV in the inactivated state and performing an energy minimization in MOE. (**A**) Structure of VSD IV (blue) and the adjacent PD I (red) highlighting the side chains of S4 gating charges (blue) and countercharges (red) of the intracellular negative cluster (INC) and the extracellular negative cluster (ENC), plus the phenylalanine (green) marking the hydrophobic constriction site (HCS). The downward movement of the S4 helix is accompanied by altered interactions formed by the S4 gating charges. In particular, R3 moved into the hydrophobic constriction site and R2 moved out of the ECN. (**B**) Close-up of the remaining inter-domain interaction between R1 with E216 in resting state 3 in Ca_V1.1e. Note that the interaction of E216 with R2 is abolished and only one single salt-bridge with R1 remains.

Table S1. Linear interaction energies of the analyzed Ca_V1 isoforms, splice-variants, and mutants.

Ca_V isoforms / mutants	Electrostatic LIE*
Ca_V1.1e	-120.4 kcal
Ca_V1.1a	<-6 kcal
Ca_V1.1e/E216A	-23.8 kcal
Ca_V1.1e/E216Q	-47.3 kcal
Ca_V1.1e/E216A/D1196N	-12.7 kcal
Ca_V1.2	-80.2 kcal
Ca_V1.3	<-6 kcal
Ca_V1.4	<-8 kcal

* LIE, linear interaction energy
